# Unconventional endosome-like compartment and retromer complex in *Toxoplasma gondii* govern parasite integrity and host infection

**DOI:** 10.1038/ncomms11191

**Published:** 2016-04-11

**Authors:** Lamba Omar Sangaré, Tchilabalo Dilezitoko Alayi, Benoit Westermann, Agnes Hovasse, Fabien Sindikubwabo, Isabelle Callebaut, Elisabeth Werkmeister, Frank Lafont, Christian Slomianny, Mohamed-Ali Hakimi, Alain Van Dorsselaer, Christine Schaeffer-Reiss, Stanislas Tomavo

**Affiliations:** 1 Center for Infection and Immunity of Lille, INSERM U 1019, CNRS UMR 8204, Institut Pasteur de Lille, Université de Lille, 59000 Lille, France; 2 Laboratory of Bio-Organic Mass Spectrometry, IPHC, CNRS UMR 7178, Université de Strasbourg, 67087 Strasbourg, France; 3 Plateforme de Protéomique et des Peptides Modifiés (P3M), Institut Pasteur de Lille, CNRS, Université de Lille, 59000 Lille, France; 4 CNRS UMR5163, LAPM, Université Joseph Fourier, Grenoble 38000, France; 5 CNRS UMR7590, Sorbonne Universités, Université Pierre et Marie Curie-Paris 6, MNHN, IRD-IUC, Paris 75005, France; 6 Bioimaging Platform, IBL, CNRS, Université de Lille, 59000 Lille, France; 7 Laboratory of Cell Physiology, INSERM U 1003, Université de Lille, 59655 Villeneuve d'Ascq, France

## Abstract

Membrane trafficking pathways play critical roles in Apicomplexa, a phylum of protozoan parasites that cause life-threatening diseases worldwide. Here we report the first retromer-trafficking interactome in *Toxoplasma gondii.* This retromer complex includes a trimer Vps35–Vps26–Vps29 core complex that serves as a hub for the endosome-like compartment and parasite-specific proteins. Conditional ablation of *Tg*Vps35 reveals that the retromer complex is crucial for the biogenesis of secretory organelles and for maintaining parasite morphology. We identify *Tg*HP12 as a parasite-specific and retromer-associated protein with functions unrelated to secretory organelle formation. Furthermore, the major facilitator superfamily homologue named *Tg*HP03, which is a multiple spanning and ligand transmembrane transporter, is maintained at the parasite membrane by retromer-mediated endocytic recycling. Thus, our findings highlight that both evolutionarily conserved and unconventional proteins act in concert in *T. gondii* by controlling retrograde transport that is essential for parasite integrity and host infection.

## Abstract (web summary)

The retromer complex is a multi-protein component of the endosomal protein sorting machinery. Here, Sangaré *et al*. identify unique features in the retromer complex of the parasite *Toxoplasma gondii*, and show that it is crucial for the biogenesis of secretory organelles in this pathogen.

## Graphical abstract

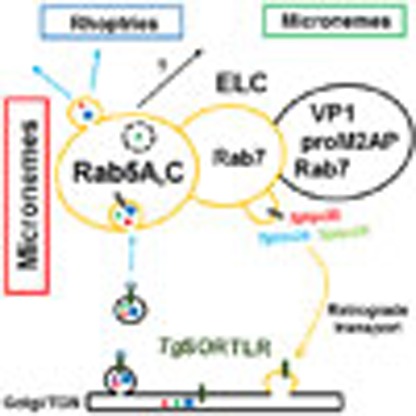

The phylum Apicomplexa comprises an ancient group of early divergent eukaryotes, including some of the most deadly pathogens of medical and veterinary importance. *Plasmodium* species are responsible for malaria, which causes as many as 700,000 deaths per year, while *Toxoplasma gondii* chronically infects up to 30% of the human population, with immunocompromised patients and pregnant women at risk for adverse outcomes, such as toxoplasmic encephalitis and spontaneous abortion, respectively^1^. *T. gondii* is considered a model system not only for its pathogenic relatives but also for intracellular parasitism and infection biology in general. *T. gondii* has common eukaryotic organelles, including the nucleus, endoplasmic reticulum and a single Golgi stack, but also specific secretory organelles named dense granules, micronemes and rhoptries that contain parasite-derived factors required for host infection. Rhoptries and micronemes are formed *de novo* during parasite replication, and this process requires significant protein and lipid trafficking through the secretory pathway.

The trafficking mechanisms employed by *T. gondii* retain several typical eukaryote components as well as evolving divergent features. Protein trafficking of this parasite is mediated by entry into a canonical endoplasmic reticulum followed by vesicle packaging through a single Golgi complex^2^,^3^. Post-Golgi protein sorting to specific organelles requires the function of dynamin-related protein B, which is involved in fission events^4^. Downstream Rab-GTPases function throughout the parasite secretory pathway^5^. *T. gondii* soluble *N*-ethylmaleimide-sensitive-factor attachment protein receptor (SNARE) proteins in docking and fusion at target membranes have also been described^6^,^7^. However, unlike in mammalian cells, *T. gondii* endoplasmic reticulum is reduced so that the nuclear envelope itself contributes to a substantial proportion of its total volume^2^. Whereas in mammalian cells hundreds of Golgi stacks occupy the perinuclear area^8^, the Golgi apparatus is limited to a single discrete structure in *T. gondii*^9^. The post-Golgi system, also named the endosome-like compartment (ELC), is involved in the trafficking of microneme proteins^10^,^11^. The ELC is decorated by the small GTPases, Rab5 and Rab7, which are typically associated with the endosomal system. Nevertheless, classical endocytosis has not yet been validated in *T. gondii*. This parasite has no lysosomes; rather the parasite harbours acidic vesicles that were thought to be precursors of the rhoptry organelles^12^. The parasite lacks most components of endosomal sorting complexes, which are known for their roles in forming multivesicular bodies that deliver ubiquitinated membrane proteins and lipids to lysosomes for degradation^3^,^13^. The machinery required for caveogenesis and caveola-dependent invaginations have not yet been identified in the parasite^14^. Furthermore, while evidence of conventional clathrin-dependent endocytosis by *T. gondii* is lacking, clathrin is present exclusively in post-Golgi compartments where its function is restricted to post-Golgi trafficking^15^, and the uptake of cytosol proteins by the tachyzoites of *T. gondii* has recently been described using an endocytosis assay^16^. However, the mechanisms underlying the events of this unconventional endocytosis in the parasite remain to be determined. Clearly, the secretory pathway of *T. gondii* can be considered a stripped-down version of the more complex trafficking machinery that characterizes higher eukaryotes. Despite this minimal trafficking machinery, the parasites actively rely on a membrane vesicle formation and transport during its intracellular lifecycle; however, to date, comparatively little is known about the mechanisms involved in trafficking pathways in *T. gondii*.

We previously reported a *T. gondii* sortilin-like receptor (*Tg*SORTLR) that regulates protein transport and is essential for apical secretory organelle biogenesis and host infection^17^. Moreover, the C-terminal tail of *Tg*SORTLR was shown to be involved in recruiting many cytosolic cargo proteins including two homologues of the core retromer components, Vps26 and Vps35 (ref. 17), which are known to regulate retrograde transport from endosomes to the *trans*-Golgi network (TGN) in yeast and mammals^18^,^19^.

Here, we report that a singular architecture with a trimer Vps35–Vps26–Vps29 core complex acts as the major endosomal cargo recycling machinery and is required for parasite integrity and more specifically for secretory organelle biogenesis and maintenance of a multiple ligand-binding transporter at the *T. gondii* membrane. Our findings provide strong evidence that the unconventional *Tg*SORTLR-containing ELC is involved in distinct mechanisms for the delivery of major retromer-dependent cargo. They also demonstrate a role for the endocytic recycling pathway in *T. gondii* pathogenesis.

## Results

### Features of the retromer interactome of *T. gondii*

**Figure 1:**
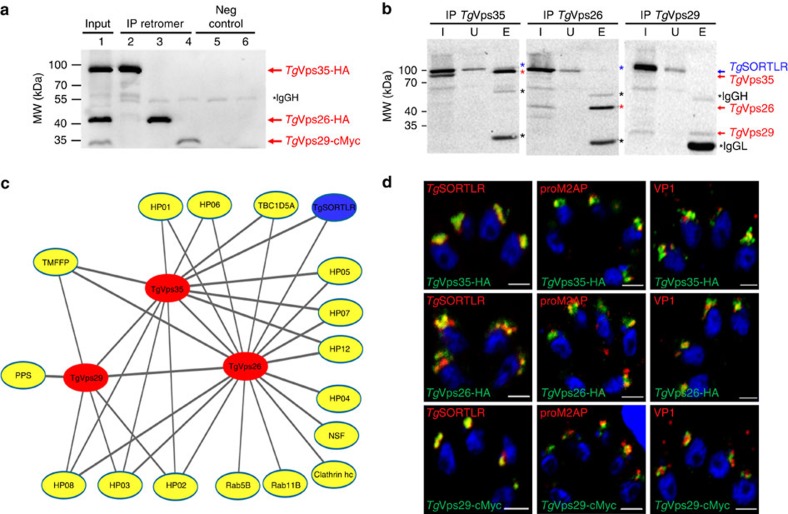
Interactome reveals cargo-selective complex and other interactors with the retromer of *T. gondii*. (**a**) Immunoblots of co-immunoprecipitates of *Tg*Vps35-HA and *Tg*Vps26-HA (designated IP retromer) probed with rabbit polyclonal specific anti-HA antibodies (lanes 2 and 3) using total detergent protein extracts from the knopck-in *Tg*Vps35-HA and *Tg*Vs26-HA parasites, respectively, and anti-HA beads. Immunoblot of co-immunoprecipitate of *Tg*Vps29-cMyc (IP retromer) probed with rabbit polyclonal anti-cMyc antibodies (lane 4) using total detergent protein extract from the knock-in *Tg*Vps29-cMyc parasites and anti-cMyc beads. Negative controls (Neg control) using total detergent protein extracts from untagged parental RH TaTi parasites incubated with anti-HA (lane 5) and anti-cMyc (lane 6) beads. Lane 1 (designated input) corresponds to equally mixed sample of all three detergent extracts containing *Tg*Vps35-HA, *Tg*Vps26-HA and *Tg*Vps29-cMyc proteins and revealed by a mixed probe containing both anti-HA and anti-cMyc antibodies. Molecular weights (kDa) of protein markers are shown on left. IgG_h_ means heavy chain of IgG. (**b**) Immunoblots of *Tg*Vps35-HA, *Tg*Vps26-HA and *Tg*Vsp29-cMyc as described in **a** probed with rat specific anti-*Tg*SORTLR antibodies. (I, input) corresponds to total detergent protein extracts from *Tg*Vps35-HA, *Tg*Vps26-HA and *Tg*Vps29-cMyc knock-in parasites, respectively; (U) unbound lysates to the anti-HA or anti-cMyc beads and (E) eluates corresponding to co-immunoprecipitates. The blots were simultaneously incubated with rat anti-*Tg*SORTLR and rabbit anti-HA or rat anti-*Tg*SORTLR and rabbit anti-cMyc antibodies. Protein markers (kDa) are also shown on left. IgG_h_ means heavy chain of IgG, IgG_L_ means light chain of IgG. (**c**) Retromer interactome was constructed by analysing the co-immunoprecipitates of *Tg*Vsp35-HA, *Tg*Vps26-HA and *Tg*Vps29-cMyc validated by immunoblotting in **a** and mass spectrometry (Supplementary Data 1). The interactome identified *Tg*Vps35, *Tg*Vps29 and *Tg*Vps26 (red) and *Tg*SORTLR (blue) in addition to the putative phosphatidylinositol synthase (PPS), transporter major facilitator family protein (TMFFP), putative N-ethylmaleimide sensitive fusion protein (NSF), multi-pass transmembrane protein (MTP), Rab5, Rab11B, Rab7-GTPase-activating protein (GAP) regulator TBC1D5A homologue and nine parasite-specific HP. (**d**) Confocal imaging of *Tg*Vps35, *Tg*Vps26 and *Tg*Vps29 that co-localize with *Tg*SORTLR, proM2AP and vacuolar protein 1 (VP1) using intracellular tachyzoites of the respective knock-in parasites stained with anti-HA or anti-cMyc antibodies followed by probing with anti-*Tg*SORTLR, anti-proM2AP and anti-VP1 antibodies, respectively. Bar, 2 μm.

To identify proteins that interact with the *T. gondii* retromer complex, we chromosomally appended an encoded hemagglutinin (HA) epitope to *Tg*Vsp35 and *Tg*Vps26. This knock-in strategy allows steady-state levels of epitope-tagged protein expression via homologous promoters. We also tagged *Tg*Vps29 identified in the parasite genome (TGME49_252490, www.toxodb.org) with a cMyc epitope as above. We performed a series of immunoprecipitation experiments under native conditions; revealing that *Tg*Vps35-HA, *Tg*Vps26-HA and *Tg*Vps29-cMyc were specifically pulled down (Fig. 1a, lanes 2–4; and Supplementary Fig. 1) using HA or cMyc-tagged protein extracts and antibodies specific to HA and cMyc, respectively. No protein signals were detected in the negative controls using naïve sera and the same protein extracts, as expected (Fig. 1a, lanes 5 and 6). In addition, immunoprecipitation of *Tg*Vps35-HA and *Tg*Vps26-HA also revealed a faint protein band corresponding to *Tg*SORTLR protein using rat antibodies anti-*Tg*SORTLR (Fig. 1b, lane 3 (E) and blue stars in left and middle panels) while immunoprecipitation of *Tg*Vps29 did not, most likely due to its low-expression level (Fig. 1b, lane 3 (E), right panel). Mass spectrometry analysis of the eluates corroborates the presence of *Tg*Vps35, *Tg*Vps26 and *Tg*Vps29 in each immunoprecipitation sample (Supplementary Data 1). Consistent with the immunoblots shown in Fig. 1b, the presence of *Tg*SORTLR was only confirmed in immunoprecipitates of *Tg*Vps35 and *Tg*Vps26 by mass spectrometry (Supplementary Data 1). To gain unbiased insight into the genuine retromer composition in *T. gondii*, we developed a quantitative approach using micro liquid chromatography-selected reaction monitoring (microLC-SRM) and stable isotope-labelled standard peptides. The absolute quantification of *Tg*Vps35, *Tg*Vps26 and *Tg*Vps29 was carried out using three proteotypic peptides per protein (Supplementary Data 2). This approach yielded a stoichiometry of ∼1:1 for *Tg*Vps35 relative to *Tg*Vps26 and 3:1 between *Tg*Vsp35 and *Tg*Vps29 (Supplementary Table 1). This stoichiometry between *Tg*Vps35 and *Tg*VPS29 is in contrast to the formation of a functional core retromer complex at a ratio of 1:1:1, as in mammalian and yeast cells^20^. However, this discrepancy may also be explained by the fact that *Tg*VPS29 may associate with *Tg*VPS35 at a much lower affinity than *Tg*VPS26, thus leading to the reduced levels of *Tg*VPS29 identified by co-immunoprecipitations. In addition, 17 retromer-interacting proteins were identified in the interactome (Fig. 1c) and ranked according to the following filtering criteria: protein common to at least two co-immunoprecipitations, absent in the control and identified with at least two unique peptides (Supplementary Data 1). Most interactors (12 out of 17) were immunoprecipitated with both *Tg*Vps35 and *Tg*Vps26 (Fig. 1c), confirming the potential of predominant *Tg*Vps35–*Tg*Vsp26 complexes in which only a fraction of *Tg*Vsp29 is bound to generate a functional retromer complex, as determined by the quantitative proteomics described in Supplementary Table 1. Functional classification by gene ontology analysis revealed that some of these interactors played roles in cell trafficking: Rab5B, an endosome marker; Rab11B, a factor essential for inner membrane complex recycling^21^; the TBC1D5A homologue, a Rab7-GTPase-activating protein that negatively regulates the core retromer function^22^; the aforementioned *Tg*SORTLR receptor^17^; and *N*-ethylmaleimide-sensitive protein, a factor involved in SNARE-dependent membrane fusion. In addition to the established binding partners, 9 out of 17 proteins are new parasite-specific proteins (that is, hypothetical proteins (HP); Fig. 1c). Confocal imaging revealed that *Tg*Vps35-HA, *Tg*Vps26-HA and *Tg*Vps29-cMyc co-localize with the ELC markers pro-microneme 2-associated protein (proM2AP), vacuolar protein 1 (VP1) and *Tg*SORTLR (Fig. 1d). We therefore conclude that the ELC defines the sub-cellular compartment where retromer-mediated vesicle recycling or retrograde trafficking operates via an endolysosomal-like system in *T. gondii*.

### *Tg*Vps35 silencing abrogates host infection by *T. gondii*

**Figure 2:**
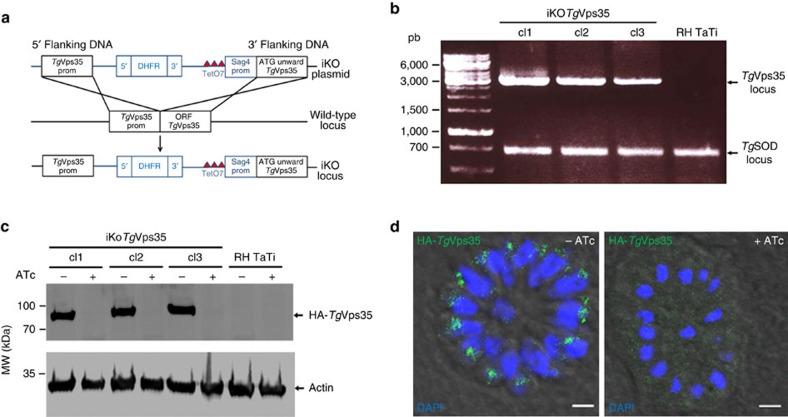
Conditional ablation of *Tg*Vps35 gene. (**a**) Schematic of the vector and experimental approach used for the conditional ablation of the *Tg*Vps35 gene. (**b**) PCR analysis of three clones with conditional disruption of *Tg*Vps35 and the parental line. Superoxide dismutase (SOD) served as the positive control. Also see the primers used for these PCR in Supplementary Table 3. (**c**) Immunoblots of the three conditional iKo*Tg*Vps35 mutants and RH TaTi parasites, which were grown in the presence or absence of ATc for 48 h, harvested and purified. Each lane refers to a total SDS-protein extract corresponding to the equivalent of 2 × 10^6^ parasites. Immunoblots were probed with anti-HA antibodies. Actin probed with specific monoclonal antibodies served as a loading control. Molecular weights (kDa) of protein markers are indicated on left. (**d**) Intracellular vacuole containing 16-daughter iKo*Tg*Vps35 mutants corresponding to one of the three clones analysed by PCR and western blots and PCR confirmed the conditional depletion *Tg*Vps35 protein (right panel) by confocal imaging after 48 h post-infection in the presence of ATc. The left panel showed the same mutant in which *Tg*Vps35 protein was detected in the endosome-like comportment (ELC) closely located to the nuclei in the absence of ATc, as expected. 4′, 6-diamidino-2-phenylindole (DAPI) was used to stain nuclei. Rabbit specific anti-HA antibodies was also used. Bar, 2 μm.

**Figure 3:**
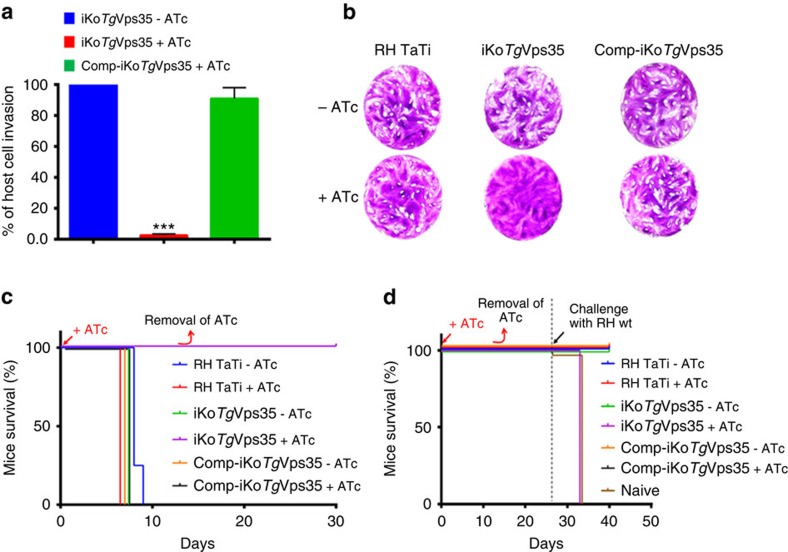
Resultant phenotypic traits of conditional disruption of *Tg*Vps35 gene. (**a**) Host cell invasion was assayed in iKo*Tg*Vps35 and complemented mutants (Comp-iKo*Tg*Vsp35) in the presence and absence of ATc. Bars indicate mean±s.d. (*n*=3, *P*<0.001 by Student's test). (**b**) Host cell lytic plaques were examined in *Tg*Vsp35-deficient mutants, Comp-iKo*Tg*Vsp35 and parental RH TaTi parasites in the presence or absence of ATc. (**c**) Survival of mice infected with lethal doses of *Tg*Vsp35-deficient mutants, Comp-iKo*Tg*Vsp35 and parental RH TaTi parasites in the presence and absence of ATc in the drinking water. The ATc was removed after 12 days and mice survival was monitored for 30 days post-infection. (**d**) Avirulent *Tg*Vps35-depleted parasites do not confer protective immunity to reinfection with lethal doses of RH wild-type parasites. ATc treatment and mice survival were monitored as above, except that sub-lethal doses of parasites were used during the primo-infection.

To establish the functional roles of *Tg*Vps35 in *T. gondii* infection, we generated conditional anhydrotetracyclin (ATc)-inducible knockout mutants (iKo*Tg*Vps35) using the strategy described in Fig. 2a. We selected three positive clones from the emerging stable parasite population and the genome editing of these clones was verified by PCR, demonstrating the perfect integration of the knockout vector at the *Tg*Vps35 locus (Fig. 2b). Following ATc treatment, we subsequently observed the disappearance of HA-*Tg*Vps35 protein by western blotting (Fig. 2c) and confocal imaging (Fig. 2d). To ascertain *bona fide* morphological phenotypes that stem from the inducible targeted disruption of the *Tg*Vps35 gene, we complemented this mutant with full-length cMyc-tagged *Tg*Vps35 (Comp-iKo*Tg*Vps35), which was introduced in the uracil phosphoribosyl transferase locus, as this gene is known to be non-essential for parasite survival^23^. The iKo*Tg*Vps35 mutants were severely impaired in their ability to invade host cells (Fig. 3a) and did not form plaques after multiple rounds of host cell invasion and lysis (Fig. 3b). Complementation of the iKo*Tg*Vps35 mutant that allows obtaining Comp-iKo*Tg*Vps35 parasite lines restored the ability of these complemented mutants to efficiently reinvade host cells (Fig. 3a), yielding normal plaque sizes in the presence of ATc similar to those of parental RH TaTi parasites (Fig. 3b). These later observations demonstrate that the lack of host cell invasion and the subsequent inability of the iKO mutants to establish several rounds of cell lysis and reinvasion are directly linked to the depletion of *Tg*Vps35 and the absence of functional retromer complex in these mutants, thus excluding pleotropic and non-specific phenotypes.

To examine the role of *Tg*Vps35 in *Toxoplasma* infection *in vivo*, mice were infected with lethal doses of iKo*Tg*Vps35, Comp-iKo*Tg*Vps35 or parental parasites followed by *Tg*Vps35 suppression *in vivo* by providing ATc in the drinking water. Strikingly, the ATc-treated mice inoculated with iKo*Tg*Vps35 survived, whereas animals inoculated with iKo*Tg*Vps35 but not treated with ATc succumbed to the infection by day 9 (Fig. 3c). Mice infected with Comp-iKo*Tg*Vps35 mutants and the parental strains succumbed to the infection regardless of the initiation of ATc treatment (Fig. 3c). It should be mentioned that RH TaTi background was genetically attenuated in virulence compared with the parental and wild-type RH strain, thus allowing challenging mice with sub-lethal parasite doses. When mice were inoculated with sub-lethal doses of these mutants or parental parasites and re-challenged with lethal doses of the wild-type parental RH strain, all iKo*Tg*Vps35-infected mice succumbed in a manner similar to the naïve primo-infected animals, whereas those infected with Comp-iKo*Tg*Vps35 and the parental strains survived (Fig. 3d). Thus, the conditional ablation of *Tg*Vps35 transformed a *T. gondii* into a complete non-lethal strain of parasites, and furthermore, infection with iKo*Tg*Vps35 parasites does not confer sterile immunity to reinfection, which is also consistent with phenotypic traits previously described for iKo*Tg*SORTLR mutants^17^.

### Retromer is essential for parasite integrity

**Figure 4:**
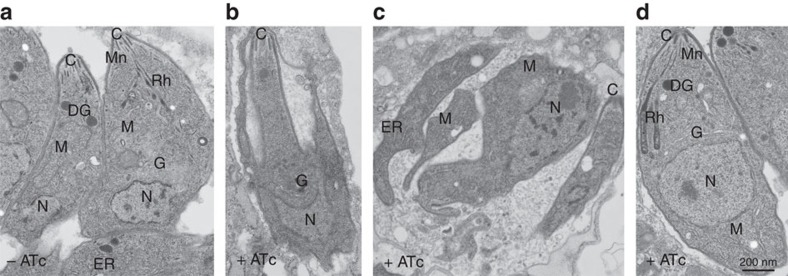
The *Tg*Vsp35 gene is essential for secretory organelle biogenesis and parasite morphology. (**a**) Transmission electron microscopy showing normal banana-shape morphology of iKo*Tg*Vps35 parasites in the absence of ATc. (**b**) An iKo*Tg*Vps35 mutant showing an aberrant morphology. (**c**) Four replicated daughter iKo*Tg*Vsp35 mutants with disorganized body shapes without rhoptries, micronemes and dense granules. (**d**) Complementation of iKo*Tg*Vps35 mutants restored normal parasite morphology with the presence of secretory organelles. C, conoid; DG, dense granules; G, Golgi; M, mitochondria; Mn, micronemes; N, nucleus; Rh, rhoptries. Bar, 200 nm.

**Figure 5:**
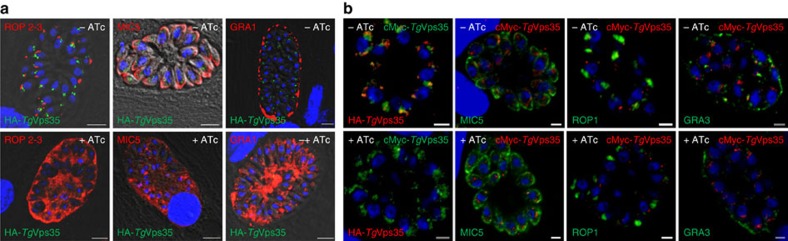
Conditional ablation of *Tg*Vps35 results in mis-sorting of ROP, MIC and GRA proteins. (**a**) Confocal immunofluorescence microscopy of ROP 2-3, MIC5 and GRA1 proteins in iKo*Tg*Vps35 mutants in the presence (lower panels) or absence of ATc (upper panels) for 48 h using specific antibodies to ROP2-3, MIC5 and GRA1 proteins (see the complete list of antibodies in Supplementary Table 5). Bar, 2 μm. (**b**) Confocal immunofluorescence microscopy of MIC5, ROP1 and GRA3 proteins in complemented iKo*Tg*Vps35 mutants in the presence (lower panels) or absence of ATc (upper panels) as above. Bar, 2 μm.

We observed that the disappearance of rhoptries peaks at 24 h of ATc treatment while micronemes were mostly affected 48 h after ATc pressure, which also corresponds to the time necessary for the complete depletion of *Tg*Vps35, as shown by western blots and confocal microscopy in Fig. 2. Following 48 h of ATc treatment, we also found a complete disorganized morphology with the marked absence of the typical banana-shaped bodies in *Tg*Vps35-depleted mutants using electron microscopy (Fig. 4b), whereas untreated iKo*Tg*Vps35 parasites appeared structurally normal with all secretory organelles (Fig. 4a). It should be mentioned that the membrane localization of the major glycosyl–phosphatidyl inositol-anchored surface antigens^24^, SAG1 and SAG3 of *T. gondii* was not impaired by the suppression of *Tg*Vps35 (Fig. 9f), indicating that the traffic to and the integrity of the parasite pellicle were not affected. This aberrant parasite morphology was confirmed in intravacuolar dividing mutants that were also devoid of rhoptries, micronemes and dense granules (Fig. 4c). Complementation of iKo*Tg*Vps35 mutants restored the ability of the parasites to form the secretory organelles *de novo* even in the ATc pressure (Fig. 4d). Using confocal microscopy, we showed that in the absence of rhoptry and microneme organelles, the ROP and MIC proteins were all mis-localized in the cytoplasm as well as in the parasitophorous vacuole of *Tg*Vps35-depleted mutants, leading to the loss of the typical apical end staining of these proteins (Fig. 5a, lower, left and middle panels, respectively). As expected, the parental parasites normally contained rhoptries and micronemes (Fig. 5a, upper, left and middle panels). In addition, the dense granule GRA1 protein staining that typically surrounds the parasitophorous vacuole (Fig. 5a, upper and right panel) was also altered in *Tg*Vps35-depleted mutants (Fig. 5a, lower and right panel). This later observation is in sharp contrast to the phenotypic traits of iKo*Tg*SORTLR mutants in which dense granule biogenesis and secretion were not affected^17^. We also confirmed that the secretory organelles were correctly localized in complemented iKo*Tg*Vps35 parasites and that the mis-sorting of ROP, MIC and GRA proteins was rescued in the presence of ATc using confocal microscopy (Fig. 5b).

**Figure 6:**
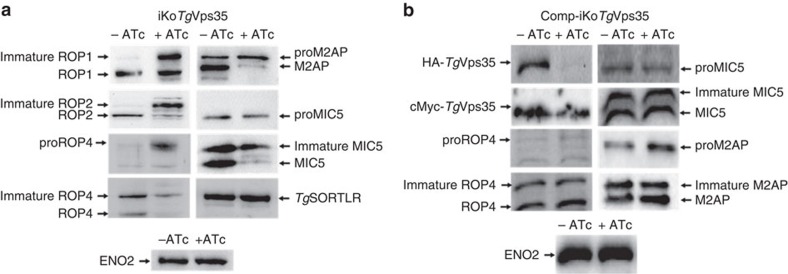
Disruption of *Tg*Vsp35 accumulates unprocessed and immature ROP and MIC proteins. (**a**) Immunoblots of iKo*Tg*Vps35 mutants probed with specific anti-ROP1, ROP2, proROP4, ROP4, M2AP, proMIC5 and MIC5 antibodies after 48 h post-infection in the presence or absence of ATc. *Tg*SORTLR and ENO2 protein levels were identical in both ATc-treated and untreated mutants. (**b**) Immunoblots of complemented iKo*Tg*Vps35 mutants probed with specific anti-ROP1, ROP2, proROP4, ROP4, M2AP, proMIC5 and MIC5 antibodies after 48 h post-infection in the presence or absence of ATc. ENO2 protein levels were identical in both ATc-treated and untreated mutants.

In wild-type parasites, formation of rhoptries and micronemes is correlated with proteolytic maturation of ROP and MIC proteins^3^,^25^. Likewise, this proteolytic maturation was defective in the *Tg*Vps35-depleted mutants compared with the parental strain, leading to the accumulation of unprocessed ROP1, ROP2, ROP4, M2AP and MIC5 (Fig. 6a and Supplementary Fig. 2). Notably, iKo*Tg*Vps35 parasites that were not treated with ATc displayed typical proteolytic maturation of the aforementioned ROP and MIC proteins (Fig. 6a and Supplementary Fig. 2). Next, we probed these blots with specific antibodies that recognized the *N*-terminal pro-peptides of ROP4 and MIC5 and found a significant accumulation of both pro-protein and immature forms in the *Tg*Vps35-deficient mutants (Fig. 6a and Supplementary Fig. 2). In contrast, processing of the receptor *Tg*SORTLR was unchanged in these mutant parasites, suggesting that neither the processing of pre-protein in the endoplasmic reticulum is impaired nor this receptor is subjected to the typical lysosomal-like degradation in the *Tg*Vps35-deficient parasites (Fig. 6a). This later behaviour of *T. gondii* lacking *Tg*Vsp35 and retromer functions differs greatly from what is normally observed in the absence of functional retromer complex in other eukaryotes in which the cargo sorting receptors such as sortilin and mannose-6-phosphate receptor are targeted to lysosomes for degradation^26^,^27^,^28^,^29^. Since the levels of the control protein, the glycolytic enzyme enolase ENO2, were similar between mutant and parental parasites, we speculated that both pro-ROP and pro-MIC specifically accumulated in iKo*Tg*Vps35 mutants as a consequence of conditional disruption of *Tg*Vps35 functions, which are clearly recovered in the presence of iKo*Tg*Vps35 complementation as cMyc-*Tg*Vps35 is able to fully restore proteolytic processing and maturation of ROP and MIC proteins under ATc pressure (Fig. 6b). Taken together, these results suggest that retromer-mediated recycling is likely required to deliver and maintain one or more proteases that process pro-ROP and pro-MIC proteins, a proteolytic processing that is a key parameter for secretory organelle formation and host infectivity by *T. gondii*.

### Secretory organelle biogenesis depends on retromer

**Figure 7:**
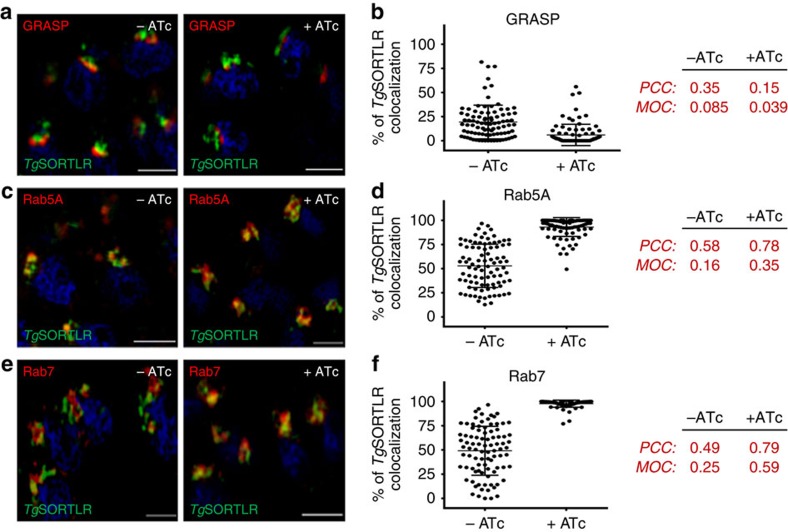
Recycling of *Tg*SORTLR is blocked in Rab5A- and Rab7-decorated ELCs. (**a**) SIM imaging of the co-distribution of *Tg*SORTLR with Golgi marker GRASP in iKo*Tg*Vps35 mutants in the absence or presence of ATc. Bar, 2 μm. (**b**) Quantification of the co-distribution of *Tg*SORTLR with Golgi marker GRASP in iKo*Tg*Vps35 mutants by SIM in the absence or presence of ATc. Bars indicated the mean *n*=90 parasites from 7 to 8 vacuoles±s.d., *P*<0.0001. (**c**) Co-distribution of *Tg*SORTLR with the marker of early endosome Rab5A in iKo*Tg*Vps35 mutants grown in the presence or absence of ATc and treated for SIM imaging as above. Bar, 2 μm. (**d**) Quantification of *Tg*SORTLR co-distributing with the marker of early endosome Rab5A in iKo*Tg*Vps35 mutants by SIM in the absence or presence of ATc. Bars indicated the mean *n*=90 parasites from 7 to 8 vacuoles±s.d., *P*<0.0001. (**e**) Co-distribution of *Tg*SORTLR with the marker of late endosome marker Rab7 in iKo*Tg*Vps35 mutants by SIM imaging as above. Bar, 2 μm. (**f**) Quantification of *Tg*SORTLR co-distributing with the marker of late endosome marker Rab7 in iKo*Tg*Vps35 mutants by SIM in the absence or presence of ATc. Bars indicated the mean *n*=90 parasites±s.d., *P*<0.0001. The Pearson correlation coefficient (PCC) and the Mander's overlap coefficient (MOC) used to quantify the degree of colocalization between the red and green fluorophores were shown on the right.

The cellular location of *Tg*Vps35, *Tg*Vps26 and *Tg*Vsp29 of the retromer complex with the ELC markers proM2AP and vacuolar protein 1 (Fig. 1d), as for Rab5 or Rab7, prompted us to investigate the outcome of *Tg*Vps35 depletion on *Tg*SORTLR, which also co-distributes in the parasite with these two small GTPases^17^. Using high-resolution structured illumination microscopy (SIM), we monitored the discrete compartments that contained *Tg*SORTLR (Fig. 7) by co-labelling Golgi apparatus marker with the Golgi reassembly stacking protein (GRASP)-RFP, the early endosome with HA-Rab5A, and the late endosome with HA-Rab7. While endogenous *Tg*SORTLR (up to 20%) colocalized with GRASP-RFP in the Golgi compartment (Fig. 7a, left panel) in the parental strain, the co-distribution drastically decreased to a marginal level in *Tg*Vps35-deficient mutants (Fig. 7a, right panel and Fig. 7b). Consequently, we observed significantly increased colocalization of endogenous *Tg*SORTLR with Rab5A and Rab7-positive ELC in *Tg*Vps35-depleted mutants (Fig. 7c,e; right panels). Quantitation of this co-distribution in *Tg*Vps35-deficient mutants indicated that 90% of *Tg*SORTLR localized with Rab5A (Fig. 7d) and that up to 99% of endogenous *Tg*SORTLR was colocalized with Rab7-positive ELC (Fig. 7f). In comparison, only 60% (Rab5A) and 50% (Rab7) of *Tg*SORTLR was colocalized with the Rab GTPase-labelled ELC in parasites that were not treated with ATc (Fig. 7d,f). Biochemical data also supported the notion that only *Tg*Vps26 interacts with Rab7 in a GTP-dependent manner, while none of these three subunits of the *T. gondii* core retromer partnered with Rab5B or Rab11B in the presence of GTP (Supplementary Fig. 3). Clearly, these data demonstrate that the retromer complex drives *Tg*SORTLR recycling from Rab7-positive ELC to the TGN, thus sustaining another round of protein transport for proper secretory organelle biogenesis.

### *Tg*HP12 is a parasite-specific retromer-associated partner

**Figure 8:**
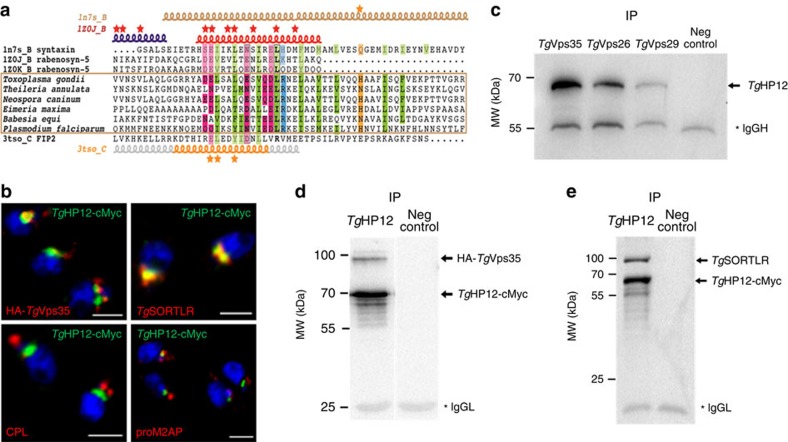
*Tg*HP12 is a novel parasite-specific retromer-associating partner. (**a**) Alignment of the helical region in *Tg*HP12 protein and in orthologues from apicomplexan parasites with human syntaxin (pdb 1n7s_B, syntaxin), rabenosyn-5 (1z0j_B and 1z0k_B), and FIP2 (pdb 3tso_C). Secondary structures are reported above and below the alignment. Stars indicate amino acids involved in Rab binding (rabenosyn-5 and FIP2) and the conserved glutamine of syntaxin that participates in the ionic central layer of SNARE complexes. Genbank identifier (gi) and N- and C-terminal limits are as follows: *Theileria annulata* (85000999, aa 239-297), *Neospora caninum* (401395596, aa 42-100), *Eimeria maxima* (557188226, aa 283-342), *Babesia equi* (510902511, aa 253-311) and *Plasmodium falciparum* (583212139, aa 375-433). Also see Supplementary Fig. 4. (**b**) Confocal immunofluorescences showing co-distribution of *Tg*HP12 protein with *Tg*Vps35 protein, and *Tg*SORTLR protein (upper panel) while no colocalization was detected with cathepsin L (CPL) or proM2AP protein. Mouse or rabbit polyclonal anti-cMyc antibodies were used in addition to rat anti-*Tg*SORTLR, rabbit anti-HA, anti-CPL and proM2AP antibodies. Bar, 2 μm. (**c**) Reverse immunoprecipitation using total detergent extract proteins from the knock-in *Tg*Vps35-HA, *Tg*Vps29-cMyc or *Tg*Vps26-HA parasites demonstrated that all three components of the core retromer complex can be pulled down by *Tg*HP12. IgG_h_, IgG heavy chain; IP, immunoprecipitations; Neg control, negative control using naïve sera; molecular weights (kDa) were shown on left. (**d**) *Tg*HP12-cMyc and *Tg*Vps35-HA proteins were concomitantly co-immunoprecipitated from total detergent protein extracts from iKo*Tg*Vps35 parasites in which *Tg*HP12-cMyc protein was expressed by knock-in strategy. The blots were probed with rabbit anti-cMyc and anti-HA. IgG_L_, IgG light chain; IP, immunoprecipitations; Neg control, negative control using naïve sera; molecular weights (kDa) were shown on left. (**e**) *Tg*HP12-cMyc and *Tg*SORTLR proteins were concomitantly co-immunoprecipitated from parasites total detergent protein extracts from iKo*Tg*Vps35 parasites in which *Tg*HP12-cMyc protein was expressed by knock-in strategy. The blots were stained with rabbit anti-cMyc and rat anti-*Tg*SORTLR. IgG_L_, IgG light chain; IP, immunoprecipitations; Neg control, negative control using naïve sera; molecular weights (kDa) were shown on left.

The conditional disruption of the *Tg*Vps35 gene strongly suggests that the retromer complex is likely involved in other functions, such as controlling parasite shape in addition to secretory organelle biogenesis. In our quest to discover other roles for the retromer complex in *T. gondii*, we sought to decipher the functions of some parasite-specific proteins also named HP that were identified in the interactome (Fig. 1c and Supplementary Data 1). Towards this goal, we searched by bioinformatics for striking sequence features that could potentially define retromer-associating proteins among these parasite-specific HP proteins. We identified a typical type I transmembrane *Tg*HP12 protein that harbours a putative coiled-coil region downstream of the transmembrane segment (Supplementary Fig. 4a). These structural features are conserved in *Tg*HP12 homologues in all tested parasites across the *Apicomplexa* phylum (Fig. 8a). Potential relationships were identified between the *Tg*HP12 helical region and two helical structures present in rabenosyn-5 and FIP2 (Supplementary Fig. 4b), which are known to be involved in the interaction with the Rab GTPases^30^,^31^, and also with the coiled-coil region of syntaxin, which shares typical heptad repeats with *Tg*HP12 (Supplementary Fig. 4b).

Because these similarities represent only short segments, statistical analysis was unable to establish a significant relationship with any of the proteins. As a result, we have not further investigated the significance of these possible structural features, but instead, we determined the molecular relationships between *Tg*HP12 and the retromer complex by knock-in *Tg*HP12-cMyc into iKo*Tg*Vsp35 mutants. These experiments revealed that *Tg*HP12 co-localizes with *Tg*Vps35-HA and *Tg*SORTLR (Fig. 8b, upper panels) but not with cathepsin L or M2AP, two markers of the ELC (Fig. 8b, lower panels), suggesting that *Tg*HP12 is a resident protein of both the Golgi and TGN compartments. Likewise, mass spectrometry was used to demonstrate that *Tg*HP12 specifically pulled down *Tg*Vps35, *Tg*Vps29 and *Tg*Vps26 in addition to *Tg*SORTLR (Supplementary Table 2). These interactions were confirmed by reverse immunoprecipitations in which *Tg*Vsp35, *Tg*Vsp29 and *Tg*Vps26 also specifically pulled down *Tg*HP12 protein (Fig. 8c). Furthermore, we confirmed that the eluates of *Tg*HP12 also contained HA-*Tg*Vps35 (Fig. 8d) and *Tg*SORTLR (Fig. 8e) by western blots. To obtain additional insight into the functions of *TgHP12*, we disrupted this gene using the CRISPR-Cas9 strategy (Supplementary Fig. 4c). We confirmed the efficient disruption of *Tg*HP12 gene, as no *Tg*HP12 protein was detected in these mutants using rat polyclonal antibodies that we specifically raised and purified against the recombinant *Tg*HP12 protein (Supplementary Fig. 4d, first and left of upper panels). As expected, this protein was normally expressed in wild-type parasites (Supplementary Fig. 4d, red, first and left of lower panels). Since no deleterious effects in rhoptries, micronemes or dense granules occurred following CRISPR-Cas9 disruption of *Tg*HP12, we conclude that this type I transmembrane protein is likely involved in functions distinct from those described for *Tg*SORTLR. In line with this hypothesis, proteomic analysis indicated that *Tg*HP12 binds to other partners (Supplementary Data 3), suggesting a possible role in alternative trafficking pathways or in the regulation of other functions as a retromer-associated partner.

### Retromer maintains a parasite transporter at the membrane

**Figure 9:**
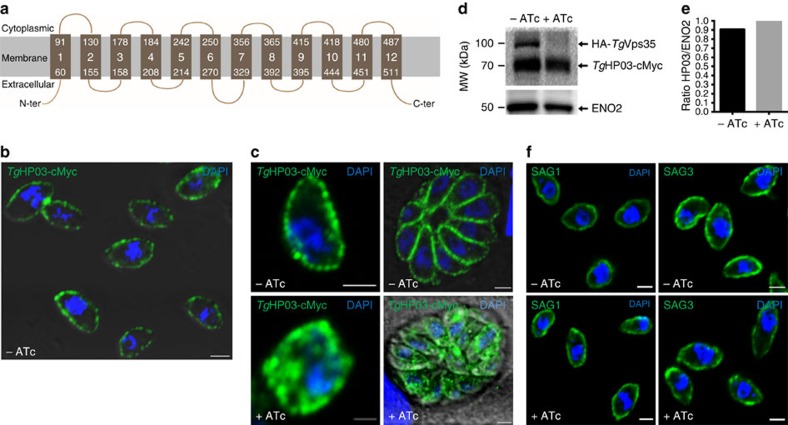
Retromer is required to maintain *Tg*HP03 in the parasite membrane. (**a**) Schematic representation of *Tg*HP03 topology inside the parasite membrane. (**b**) Confocal imaging of extracellular parasites with knock-in *Tg*HP03-cMyc in the iKo*Tg*Vps35 mutants. Immunofluorescence assay (IFA) was performed in the absence of ATc and detergent permeabilization. Nuclei of the parasites as stained with 4′, 6-diamidino-2-phenylindole (DAPI; blue). Bar, 2 μm. (**c**) Magnified image of an extracellular parasite expressing *Tg*HP03-cMyc protein in the iKo*Tg*Vps35 mutants grown in the absence of ATc and detergent permeabilization (upper, left panel); intracellular parasites expressing *Tg*HP03-cMyc protein in the iKo*Tg*Vps35 genetic background. IFA was performed in the absence of ATc and in the presence detergent permeabilization (upper, right panel); magnified image of an intracellular parasites expressing *Tg*HP03-cMyc protein in the iKo*Tg*Vps35 genetic background in the presence of ATc and detergent permeabilization (lower, left panel) and intracellular parasites expressing *Tg*HP03-cMyc protein in the iKo*Tg*Vps35 genetic background in the presence of ATc and detergent permeabilization (lower, right panel). Bar, 2 μm. (**d**) Immunoblots of parasites expressing *Tg*HP03-cMyc protein in the knock-in iKo*Tg*Vps35 mutants grown in the presence or absence of ATc. ENO2 was used as a loading control. Molecular weights (kDa) were shown on left. (**e**) Quantification of *Tg*HP03 levels in these parasites expressing *Tg*HP03 protein in the knock-in iKo*Tg*Vps35 mutants that were grown in the absence or presence of ATc. (**f**) The surface localization of glycosyl–phosphatidyl inositol (GPI)-anchored SAG1 and SAG3 were determined in iKo*Tg*Vps35 mutants in the presence or absence of ATc using monoclonal antibodies specific to SAG1 and SAG3. Bar, 2 μm.

The generation of iKo*Tg*Vps35 mutants that are deficient in retromer-mediated transport allows us to investigate the recycling mechanisms that deliver and maintain transmembrane proteins at the parasite membrane. We also used bioinformatics to search for candidate multi-spanning transmembrane proteins among the HP identified in the retromer interactome. We discovered that *Tg*HP03 exhibits the topology and positions of 12 transmembrane helices that were predicted by Phyre2 alignments^32^ to align with known three-dimensional structures of several members of the major facilitator superfamily (MFS), suggesting that *Tg*HP03 may belong to this superfamily (Fig. 9a). These membrane transporters facilitate movement of a wide range of small substrates such as metabolites, oligosaccharides, amino acids, oxyanions and drugs that were all transported by MFS across the cell membranes^33^. Even though a significant relationship with the MFS can be established, no direct link with one specific MSF member was determined by bioinformatics. To assess the functional links between *Tg*HP03 and the retromer complex, we first examined its sub-cellular localization by detecting a cMyc epitope-tagged version in the iKo*Tg*Vps35 mutants. In the absence of ATc, *Tg*HP03-cMyc displayed homogenous membrane staining on both extracellular (Fig. 9b,c, left upper panel) and intracellular (Fig. 9c, right upper panel) parasites, as expected for transmembrane transporters. *Tg*Vps35 suppression by ATc led to a mis-localization and enhanced accumulation of *Tg*HP03 in intra-cytoplasmic vesicular structures in both extracellular (Fig. 9c, left lower panel) and intracellular mutants (Fig. 9c, right lower panel), thus leading to a decrease of *Tg*HP03 at the parasite surface. Since ATc treatment did not affect the levels of *Tg*HP03-cMyc protein (Fig. 9d,e), it is likely that no *Tg*HP03 protein degradation may occur on suppression of *Tg*Vps35-mediated endocytic recycling. This observation is also consistent with the absence of *Tg*SORTLR degradation following suppression of *Tg*Vps35 (Fig. 6a). Again, the accumulation of mis-sorted cargo in intra-cytoplasmic vesicles in *T. gondii* is also in sharp contrast to transmembrane transporter degradation occurring via lysosomes in mammalian cells^34^. Interestingly, we found that the surface localization of the glycosyl–phosphatidyl inositol-anchored major surface antigens, SAG1 and SAG3 of *T. gondii* was not influenced by the suppression of *Tg*Vps35 (Fig. 9f), suggesting that only the sorting of multi-spanning transmembrane proteins may be influenced by retromer-dependent endocytic recycling. Together, our data identify a role for *Tg*Vsp35 in the endosome-to-plasma membrane sorting of multi-spanning transmembrane transporter cargo and provide the first evidence for the mechanistic role of *Tg*Vsp35 in the process linked to retromer-dependent endocytic recycling.

## Discussion

Nascent apical organelles require proficient cell trafficking to fulfil their critical role during invasion and intracellular development of *T. gondii*. Here, we show that retromer-mediated recycling is essential for secretory organelle biogenesis, parasite morphology and maintenance of a transmembrane transporter at the parasite membrane. The retromer complex was first identified in yeast and mammals as a heteropentameric complex typified by a cargo-selective complex that was built around the Vps35–Vps29–Vps26 trimer and a dimer of different sorting nexins^28^,^35^,^36^,^37^,^38^. Our previous findings that *Tg*SORTLR receptor is involved in the recruitment of Vps26 and Vps35 homologues prompted us to investigate how the retromer complex regulates retrograde transport as well as other functions in *T. gondii*. Towards this goal, we characterized the retromer interactome that includes *Tg*Vps35, *Tg*Vps29 and *Tg*Vps26 proteins. We failed to identify homologues of nexins (SNX) in the immunoprecipitates of *T. gondii* using all three retromer components, confirming that no SNX proteins can be pulled down in association with the retromer cargo-selective trimer^39^. However, we also noticed the paucity of nexin-like proteins in the genome databases of *T. gondii*, and this may also explain our inability to pull down recognizable nexin-like proteins in the parasite. Rather, we identified a singular retromer complex typified by the *Tg*Vps35–*Tg*Vps29–*Tg*Vps29 trimer. This complex interacts with *Tg*SORTLR, *N*-ethylmaleimide-sensitive protein, Rab5B, Rab11B and the TBC1D5A homologue, a member of the Rab7-GTPase-activating proteins known to negatively regulate the retromer complex through Rab7 dissociation from endosomal membranes^22^. However, we cannot rule out the possibility that some parasite-specific proteins may act as SNX and Bin/Amphyphysin/Rvs domain-containing proteins that are required for recruitment of the retromer complex to endosome membranes, membrane curvature and tubulation^27^,^35^. Furthermore, we demonstrated that *Tg*SORTLR recycling from the ELC to the TGN is severely compromised in *Tg*Vps35 mutants, suggesting that the retromer complex functions in endocytic recycling in *T. gondii.* In contrast to yeast and human cells in which Rab7–Vps35 interaction is necessary for binding to endosome membranes^37^,^38^, we found an unconventional *Tg*Rab7–*Tg*Vps26 interaction, a singular feature previously described in *Entamoeba histolytica*^40^.

In addition, we describe additional evidence for the phenotypic links between an absence of proteolytic processing of ROP/MIC proteins, mis-sorting of their unprocessed forms, and loss of rhoptry and microneme organelles. Our hypothesis is that *Tg*SORTLR, in addition to acting as a sorting receptor for ROP/MIC proteins, delivers proteases, which are involved in the processing of pro-proteins, to secretory organelles.

The retromer interactome also includes several parasite-specific proteins. We first described *Tg*HP12 as a novel type I transmembrane protein with helical regions that share potential similarities with Rab-binding or SNARE-like domains. We showed that *Tg*HP12 localizes to the TGN and physically interacts with the core retromer complex *Tg*Vps35–*Tg*Vps29–*Tg*Vps26 as well as with *Tg*SORTLR. Genetic ablation of *Tg*HP12 using CRISPR-Cas9 indicates that its function is neither essential nor related to secretory organelle biogenesis. Instead, proteomic analyses revealed other unrelated secretory organelle proteins whose functions remain to be determined. Our current study also describes for the first time in apicomplexan parasites, the endocytic recycling of the multi-pass transmembrane transporter *Tg*HP03, which belongs to the MFS, a class of membrane transport proteins that facilitate movement of small solutes across cell membranes such as drugs, metabolites, oligosaccharides and amino acids in response to chemiosmotic gradients^41^. *Tg*HP03 accumulates in endocytic vesicles that are detected in the cytoplasm of *Tg*Vps35-deficient mutants, indicating that maintenance of *Tg*HP03 at the surface of *T. gondii* requires endocytic recycling from endosomes to the plasma membrane. Clearly, our study underscores the wide range of possible cargo molecules that are recycled by the retromer complex in light of the numerous identified transmembrane proteins that require future examination. A global analysis of retromer-mediated cargo *per se* will aid the delineation of the diverse metabolite and metal ion transporters required to maintain parasite nutrient homeostasis and intracellular replication.

It is also worth noting that the absence of *Tg*Vps35 has negatively impacted the biogenesis of dense granules and body morphology in addition to that of rhoptries and micronemes, whereas *Tg*SORTLR functions are restricted to rhoptry and microneme formation^17^. This disorganization of parasite body shape in the absence of *Tg*Vsp35 suggests a possible additional role of the retromer in regulating cytoskeleton and endosome functions as previously described in mammalian cells^42^,^43^. However, the sorting nexins that are involved in this process in mammalian cells are not presently identified in *T. gondii*. Alternatively, we cannot rule out the possibility that the parasite-specific HP or other retromer-associated proteins could play this novel function that controls body morphology in *T. gondii*.

**Figure 10:**
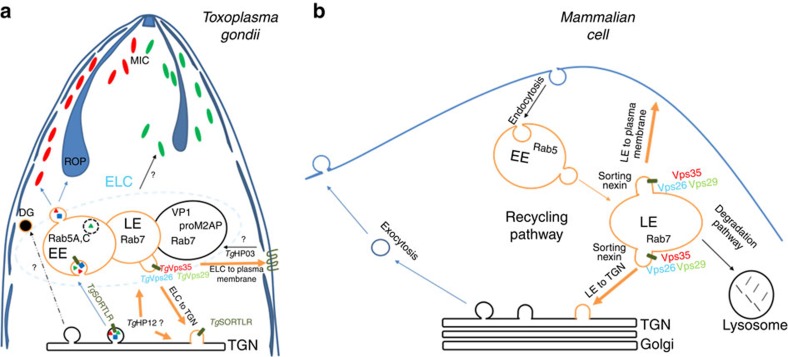
Model of retromer-mediated recycling of diverse sorting cargoes in *T. gondii versus* mammalian cells. No recognizable sorting nexins, Bin/Amphyphysin/Rvs (BAR)-containing domain proteins or lysosomal-like protein degradation have been identified in *T. gondii* (**a**), in contrast to the situation in mammalian cells (**b**). Instead, the retromer-dependent recycling is essential for secretory organelle formation and parasite shape. We propose a model suggesting that Rab7 is the key small GTPase, which is involved in the endocytic recycling of *Tg*SORTLR from endosomes to TGN for another round of ROP and MIC transport and secretory organelle biogenesis. We further raised the possibility that in contrast to mammalian cells, *T. gondii* lysosomal-like organelles only promote proteolytic maturation of proteins destined to secretion and that the endosomal system is adapted for organellar discharge of virulence-like factors required for the intracellular lifestyle of the parasite. Moreover, we provide the first evidence that a multiple ligand transmembrane transporter *Tg*HP03 is maintained at the surface of *T. gondii* through endocytic recycling from endosomes to the plasma membrane. BAR, Bin/Amphyphysin/Rvs; MIC, microneme; ROP, rhoptry; *Tg*HP03, *T. gondii* hypothetical protein 03.

In conclusion, we demonstrated that the retromer complex of *T. gondii* is a major endosomal recycling hub required for sorting different cargo proteins that regulate diverse functions vital for parasite survival, as indicated in the our model presented in Fig. 10. This model also supports the notion that the retromer complex drives *Tg*SORTLR recycling from Rab7-positive ELC to the TGN, thus sustaining another round of protein transport for proper secretory organelle biogenesis (Fig. 10a). In contrast to higher eukaryotes, in which an increase of lysosome degradation of the glucose transporter and other cargo sorting proteins, sortilin and mannose-6-phosphate receptor occurred in the absence of Vps35 (refs 28, 34), the disruption of *Tg*Vsp35 did not affect *Tg*SORTLR and *Tg*HP03 degradation. This later observation also supports our model that in contrast to mammalian cells (Fig. 10b), *T. gondii* lysosomal-like organelles^11^,^44^,^45^, only promote proteolytic maturation of proteins destined to secretion and that the endosomal system is adapted for organelle biogenesis and discharge of factors required for the intracellular lifestyle of the parasite (Fig. 10a). Our findings are expected to advance our understanding of endocytic recycling by *T. gondii*, highlighting the identity of unconventional endosomal factors, and this new knowledge may ultimately reveal new targets for managing toxoplasmosis.

## Methods

### Growth of host cells and parasite strains

*T. gondii* RH wild-type strain and its derivative RHΔku80 (ref. 46) or RH TaTi (ref. 47) strains were grown in human foreskin fibroblasts (HFF; from ATCC, USA) as described^17^. Plaque size assays were performed using HFF cells infected with 250 parasites in the presence or absence of 1.5 μg ml^−1^ of ATc for 6 days followed by crystal violet staining. All stained coverslips were imaged with an Axioplan microscope (Zeiss).

### Generation of knockout and knock-in *T. gondii* strains

All primers used for PCR are listed in Supplementary Table 3. The conditional iKo*Tg*Vps35 mutants were generated in the RH TaTi strain using the pG13-D-T7S4 plasmid, which contains 2 kb of 5′ and 3′ genomic DNA and the dihydrofolate reductase (DHFR) gene for pyrimethamine selection. After transfection of 5 × 10^6^ parasites with 50 μg of linear plasmid, a stable line was cloned by limiting dilution. For complementation, iKo*Tg*Vps35 mutants were transfected with 50 μg of plasmid containing the full-length cMyc-tagged *Tg*Vps35 gene inserted into the uracil phosphoribosyl transferase locus. Stable transgenic and cloned parasites were selected with 5 μM 5-fluoro-2′-deoxyuridine. Transgenic *Tg*Vps35-HA, *Tg*Vps26-HA and *Tg*Vps29-cMyc parasites were generated with a knock-in strategy with DNA fragments of 2.1, 2.1 and 1.8 kb, respectively, cloned upstream of the stop codon from TGGT1_242660, TGGT1_263500 and TGGT1_252490 genes. DNA sequences were cloned into the pLIC-HA-DHFR and pLIC-cMyc-DHFR plasmids^46^. Tachyzoites (5 × 10^6^ parasites) of the RHΔKu80 strain were transfected with 25 μg of linearized plasmids. Transgenic parasites expressing cMyc-tagged *Tg*HP03 (TGGT1_240810) and *Tg*HP12 (TGGT1_294220) were also generated as described above. All plasmids used in this study are listed in Supplementary Table 4.

### Transient transformation and Cas9-mediated gene disruption

The plasmid pTOXO_Cas9-CRISPR (see map in Supplementary Fig. 4, panel **c**) corresponds to pUC57 carrying the C-terminally HA/GFP tagged *S. pyogenes Cas9* gene^48^ fused to 2 nuclear localization sequences expressed under the control of the TUB8 promoter as well as the *Tg*U6 promoter driving the gRNA. Twenty mers-oligonucleotides corresponding to specific gene of interest were cloned using Golden Gate strategy^49^. The ccdB positive-selection marker acts by killing the background of cells with no cloned DNA. The plasmid was synthesized and fully sequenced by GenScript (Singapour).

### Confocal microscopy

Extracellular or intracellular parasites were fixed with 4% paraformaldehyde in phosphate-buffered saline for 20 min and processed as described^17^,^50^ using the indicated antibodies. The sources, origins and dilutions of all antibodies used for immunofluorescence assays were listed in Supplementary Table 5. Samples were observed with a Zeiss Confocal or Apotome microscope, and images were processed using ZEN software (Zeiss).

### Structured illumination microscopy

SIM was used to obtain high-resolution images using an ElyraPS1 microscope system (Zeiss) with a × 100 oil immersion lens (alpha Plan Apochromat × 100, NA 1.46, oil immersion). The resolution was measured using beads with a diameter of 100 nm (Tetraspeck multicolor). The point spread function was calculated using the metroloJ plugin (ImageJ, NIH), which gave an *x*–*y* resolution of 125 nm and a *z* resolution of 500 nm. A voxel size of 0.050 × 0.050 × 0.150 μm^3^ was used for the measurement. Three lasers (405, 488 and 561 nm) were used for excitation. SIM images were acquired with an EMCCD camera (Andor Technology Ltd, UK) and processed with ZEN software, exposure times varied between 100 and 120 ms. Three-dimensional images were generated using a z-step of 150 nm (total thickness ∼5 μm), while reconstructions and co-distributions were determined with IMARIS software (Bitplane). For co-distribution analysis, we used the colocalization module implemented in the IMARIS software. To process the images, we first applied a threshold for each channel (threshold adapted according to the labelling). For the Alexa488 channel, we used a threshold fixed at 15,000. For the red channel, the threshold varied between 8,000 and 25,000, depending on the dye used. The percentage (%) of volume B (green channel) above the threshold that colocalized (colocalized volume B above threshold/total volume B above threshold) and thresholded Mander's coefficient B and Pearson coefficient were calculated. The acquisition was performed sequentially using 43HE, 38HE and BP 420–480 Zeiss filter sets, and 15 frames were acquired to reconstruct one image (five rotations × three phases, with a SIM grating period of 51 μm for the blue channel, 42 μm for the green channel and 34 μm for the red channel). Beads with a diameter of 100 nm were imaged to measure the chromatic misalignment of our system (fit procedure by the ZEN software), and these parameters enabled further correction of the alignment on each acquired multi-channel stack. Image reconstruction was achieved using ZEN software with the following parameters: Noise Level -4, Sectioning 99-83-83 and Frequency Weighting 1.

Specifically, iKo*Tg*Vps35 mutants transiently transfected were treated with ATc or left untreated for 24 h or 48 h, fixed and stained with primary specific antibodies and either Dylight594- or Alexa488-conjugated secondary antibodies. Forty eight hours of ATc treatment corresponds to the time point where optimal effects on rhoptry and microneme biogenesis were observed and expression of *Tg*Vps35 protein was completely ablated as demonstrated by western blots and IFA. Therefore, all other experiments were then performed at this time point in this study. For GRASP-RFP expressing parasites, the fluorescence signal was directly visualized by SIM. Colocalization was quantified as overlap between green and red images using the IMARIS software for 90 intracellular parasites from 7 to 8 vacuoles.

### Electron microscopy

For transmission electron microscopy, intracellular tachyzoites of iKo*Tg*Vps35, complemented mutants, or parental parasites were fixed overnight at 4 °C with 2.5% glutaraldehyde/4% paraformaldehyde prepared 0.1 M cacodylate buffer, cells were fixed in 2.5% glutaraldehyde prepared in 0.1 M cacodylate buffer and post-fixed in 1% osmium tetroxide in the same buffer. After acetonitrile dehydration, the pellet was embedded in Epon. Ultrathin sections (90 nm) were cut using a Leica UC7 ultramicrotome and collected on 150 mesh hexagonal barred copper grids. After staining with 2% uranyl acetate prepared in 50% ethanol and incubation with a lead citrate solution, sections were observed on a Hitachi H-600 transmission electron microscope at 75 kV accelerating voltage.

### Host cell invasion assays

The conditional iKo*Tg*Vps35 mutants were treated with ATc or left untreated for 48 h and then used to infect HFF cells for 1 h at 37 °C. Twenty-four hours post-infection, coverslips were fixed and processed for immunofluorescence assays. The intracellular parasites were labelled with anti-SAG1 antibodies, and the nucleus was stained with 4′, 6-diamidino-2-phenylindole. For each condition, 400 microscopic fields (× 40) were observed. The number of SAG1-positive parasites was compared with the number of cells stained with 4′, 6-diamidino-2-phenylindole. The ratio parasites/cells are presented as mean values±s.d. from three independent experiments.

### Mouse infectivity studies

A group of 4 female BALB/C mice (6–8-week-old, from Janvier Labs, France) were intraperitoneally injected with 2 × 10^6^ tachyzoites of RH TaTi, iKo*Tg*Vps35 or Comp-iKo*Tg*Vps35 parasites. To suppress *Tg*Vps35 expression, the drinking water was supplemented with 0.2 mg ml^−1^ of ATc and 5% of sucrose. As controls, a group of four mice was also infected with the same parasite lines without ATc. Survival was monitored over 4 weeks. For vaccination assays, BALB/C mice were infected by intraperitoneal injection with 3 × 10^2^ tachyzoites of RH TaTi, iKo*Tg*Vps35 or Comp-iKo*Tg*Vps35 parasites followed by ATc treatment or not. After 4 weeks, the survived mice were re-challenged with 1 × 10^3^ tachyzoites of wild-type RH strain and survival monitored for 30 days. All animal experiments were performed following the guidelines of the Pasteur Institute of Lille animal study board, which conforms to the Amsterdam Protocol on animal protection and welfare, and Directive 86/609/EEC on the Protection of Animals Used for Experimental and Other Scientific Purposes, updated in the Council of Europe's Appendix A (http://conventions.coe.int/Treaty/EN/Treaties/PDF/123-Arev.pdf). The animal work also complied with the French law (no. 87-848 dated 19 october 1987) and the European Communities Amendment of Cruelty to Animals Act 1976. All animals were fed with regular diet and all procedures were in accordance with national regulations on animal experimentation and welfare authorized by the French Ministry of Agriculture and Veterinary committee (permit number: 59-009145).

### Co-immunoprecipitation and western blots

Tachyzoites (1 × 10^9^ parasites) from *Tg*Vps35-HA, *Tg*Vps26-HA, *Tg*Vps29-cMyc and RHΔku80 strains were lysed with 10 mM HEPES, pH 7.9; 1.5 mM MgCl_2_; 10 mM KCl; 0.5 mM dithiothreitol (DTT); 0.1 mM EDTA; 0.65% NP40; 0.5 mM phenylmethanesulfonylfluoride (PMSF); and a cocktail of protease inhibitors (Sigma Aldrich). After 30 min on ice, the lysates were centrifuged at 21, 693*g* for 30 min at 4 °C, and the supernatants were then incubated with anti-HA or anti-cMyc agarose beads (Thermo Pierce) overnight at 4 °C under rotating shaker. After five washings with 10 mM Tris, pH 7.5; 150 mM NaCl; 0.2% Triton X-100; 0.5 mM PMSF, and a final washing with 62.5 mM Tris, pH 6.8, immunoprecipitates were eluted with Laemmli buffer (0.2% SDS, 100 mM DTT, and 10% sucrose). These samples were analysed by SDS–polyacrylamide gel electrophoresis (SDS–PAGE) followed by silver staining before proteomics analyses. For western blots, parasites were lysed with Laemmli buffer, and lysate proteins were separated on 12% acrylamide gels and processed for immunoblotting using primary specific antibodies and then secondary antibodies conjugated to alkaline phosphatase (Thermo Pierce). All primary antibodies used for western blots were listed in Supplementary Table 5. The blots were imaged using ChemiDoc XRS^+^ (Bio-Rad).

### Liquid chromatography coupled to mass spectrometry

For liquid chromatography and mass spectrometry performed using Q-Exactive mass spectrometer (Thermo Scientific, Bremen, Germany), each sample (30 μl) was denatured with Laemmli sample buffer and loaded onto one-dimensional SDS–PAGE (12%), stained with colloidal Coomassie G-250 (Bio-Rad, Hercules, CA) and six slices per sample were excised for reduction and alkylation. For this step, gel slices were cut in small pieces (1 mm^3^) and the staining of gel pieces were removed thrice with 120 μl of a mixture of 50/50 (v/v), 25 mM ammonium bicarbonate (NH_5_CO_3_)/acetonitrile for 10 min. In-gel reduction and alkylation of protein disulfide bonds were performed, respectively, with 100 μl of 10 mM of DTT in 50 min at 57 °C and 100 μl of 50 mM of iodoacetamide (IAM) for 30 min at room temperature. After a washing step with 120 μl of 25 mM NH_5_CO_3_ and the dehydration step with 100 μl acetonitrile for 5 min, an in-gel digestion was performed on each sample with 0.07 μg of sequencing grade porcine trypsin (Promega, Madison, WI) for 16 h at 37 °C. The peptide were extracted thrice from gel with a mixture of 60/40/0.1 (v/v/v), acetonitrile/25 mM of NH_5_CO_3_ (v/v) and 0.1% formic acid. The extracted solution were then dried with vacuum centrifuge (Uniequip GmbH, Munich, Germany) and resuspended in 10 μl of water containing 0.1% formic acid.

Each extracted solution of 3 μl was injected into the Ultimate 3,000 RSLC nano- System (Dionex, Thermo Scientific) through a trap column (Acclaim PepMap, 5 mm × 300 μm inner diameter, C18, 5 μm, 100 Å; Dionex) at 5 μl min^−1^ with water containing 0.1% formic acid and 2% acetonitrile. After 5 min, the trap column was set on-line with analytical column (Acclaim PepMap RSLC, 15 cm × 75 μm inner diameter, C18, 2 μm, 100 Å; Dionex, Sunnyvale, CA). The elution was carried out by applying mixture of solvent A (HPLC grade water with 0.1% formic acid) and solvent B (HPLC grade acetonitrile with 0.1% formic acid) at the flow rate of 300 nl min^−1^. The separations were performed by applying a linear gradient of 2–40% solvent B over 38 min followed by a washing step (5 min at 70% solvent B) and an equilibration step (11 min at 2% solvent B).

The eluted peptides were analysed by a Q-Exactive mass spectrometer. For ionization, a nanospray Flex Ion Source was used with a voltage of 1.9 kV, and the capillary temperature was 275 °C. Full MS scans were acquired in the Orbitrap mass analyser over *m*/*z* 300-3,500 range with resolution of 70,000 at *m*/*z* 200. The target automatic gain control value of 1 × 10^6^ was used with a maximum allowed injection time (Maximum IT) of 250 ms. For MS/MS, an isolation window of 2 *m*/*z* was utilized. Ten most intense peaks (TopN) with charge state between 2 and 6 were selected for fragmentation in the higher-energy collisional induced dissociation cell with normalized collision energy of 35. The tandem mass spectra were acquired over *m*/*z* 200-2,000 range in the Orbitrap mass analyser with resolution 35,000 at *m*/*z* 200 and an automatic gain control of 2 × 10^5^. The ion intensity selection threshold was 6.7 × 10^4^, and the maximum injection time was 150 ms. The dynamic exclusion time was 10 s and. the total run time was 60 min. All these systems were fully controlled by Thermo Xcalibur 3.0 (Thermo Fisher Scientific).

All data files (*.raw) collected were processed with a specific workflow designed in Proteome Discoverer 1.4 (Thermo Fisher Scientific). MS/MS data was interpreted using two search engine Mascot (version 2.4.1, Matrix Science, London, UK) and Sequest HT (Thermo Fisher Scientific). Searches were performed against *T. gondii* (TGVEG, TGME49 and TGGT1 stain) protein sequences downloaded from www.toxodb.org at the 11th December 2014 (18,954 entries). The Mascot ion score were >20 and Sequest HT XCorr >1.5. The target-decoy database search allowed us to control and to estimate the false positive identification rate^51^.

### Nanoscale liquid chromatography and tandem mass spectrometry

For nanoscale liquid chromatography coupled to tandem mass spectrometry (NanoLC-MS/MS), samples were electrophoresed onto 12% SDS–PAGE and stained overnight with colloidal Coomassie Brilliant Blue. Gel bands were manually excised, reduced 1 h at 60 °C by adding DTT to a final concentration of 10 mM and alkylated by adding iodoacetamide to a final concentration of 40 mM. An overnight digestion was performed by adding trypsin (Promega). Tryptic peptides were extracted (60% acetonitrile, 0.1% HCOOH) before mass spectrometry analyses. NanoLC-MS/MS analyses were performed on three different systems: nano-ACQUITY Ultra-Performance-LC system (UPLC; Waters, Milford, MA, USA) hyphenated to either Q-TOF Impact HD or MaXis 4G (Bruker Daltonics, Bremen, Germany) and a nanoLC-Chip/Cube (Agilent Technologies, Palo Alto, CA, USA) hyphenated to an ion trap amaZon (Bruker Daltonics). For maXis 4G and amaZon analysis, methods used were previously described^52^ with slight modifications. For Impact HD analysis, peptides were first trapped on a 0.18 × 20 mm^2^, 5 μm Symmetry C18 pre-column (Waters), and then separated on an ACQUITY UPLC BEH130 C18 column (Waters), 75 μm × 250 mm with 1.7 μm particle size. The solvent system consisted of 0.1% HCOOH in water (solvent A) and 0.1% HCOOH in acetonitrile (solvent B). Trapping was performed for 3 min at 5 μl min^−1^ with 99% A and 1% B. Elution was performed at a flow rate of 450 nl min^−1^, using a 1–35% gradient (solvent B) over 30 min at 60 °C.

The mass spectrometer was equipped with a Captive Spray source and a nano-Booster operating in positive mode, with the following settings: source temperature was set at 150 °C while drying gas flow was at 3 l min^−1^. The nano-electrospray voltage was optimized to −1,300 V. External mass calibration of the time-of-flight (TOF) was achieved before each set of analyses using Tuning Mix (Agilent Technologies) in the mass range of 322–2,722 *m*/*z*. Mass correction was achieved by recalibration of acquired spectra to the applied lock masses hexakis (2,2,3,3,-tetrafluoropropoxy) phosphazine ([M+H]^+^=922.0098 *m*/*z*)].

For tandem MS experiments (CID), the system was operated with fixed cycle time of 3 s in the range of 150–2,200 *m*/*z*. MS/MS scan speed was monitored in function of precursor intensity from 4 to 25 Hz. Ions were excluded after acquisition of one MS/MS spectra and the exclusion was released after 1 min. The complete system was fully controlled by Hystar 3.2 (Bruker Daltonics).

### Bioinformatics and protein identification

Mass data collected during nanoLC-MS/MS analyses were processed, converted into ‘.mgf' files with Data Analysis 4.0 (Bruker Daltonics) and interpreted using MASCOT 2.5.1 algorithm (Matrix Science, London, UK) running on a local server. Searches were performed without any molecular weight or isoelectric point restrictions against an in-house generated protein database composed of protein sequences of *T. gondii* (ToxoDB database, October 2014) and known contaminant proteins such as human keratins and trypsin. All proteins were concatenated with reversed copies of all sequences (49,328 entries) with an in-house database generation toolbox https://msda.unistra.fr^53^. Trypsin was selected as enzyme, carbamidomethylation of cysteine (+57 Da) was set as fixed modification, oxidation of methionine (+16 Da) were set as variable modification and both precursor and fragment mass tolerances were adapted according to instrumental mass accuracy. Mascot results were loaded into the Proline software (Proline Studio Release 1.0) and filtered to obtain a false discovery rate of <1%.

### Absolute quantitation using LC-SRM

For microLC-SRM assay, three proteotypic peptides per targeted protein (*Tg*Vps35, *Tg*Vps26 and *Tg*Vps29) were selected. A total of nine high-purity isotopically labelled equivalent peptides were purchased (AQUA peptides, Thermo Fischer Scientific, Bremen; Germany). Previous nanoLC-MS/MS analyses afforded a representative MS/MS spectrum for each peptide. Four to six transitions were monitored for both endogenous and heavy-labelled peptides. Thus, a total of 78 transitions corresponding to 20 precursors and 3 proteins were measured. For the lower limits of quantification and detection determination, a dilution series of the labelled peptides was realized at different concentrations in a mixture containing all tagged proteins and injected in triplicate on a QQQ-6490 triple quadrupole mass spectrometer (Agilent Technologies). The area under curve of the three best transitions per peptide were summed and drawn versus the peptide concentration. Two calibration curves were drawn *per* peptide: high calibration curve (15 fmol μl^−1^–238 fmol μl^−1^) and low-calibration curve (2 fmol μl^−1^–30 fmol μl^−1^). We evaluated the lower limits of quantification and the lower limits of detection determination by applying recognized definitions^54^.

For the SRM analyses, samples were electrophoresed onto 4% SDS–PAGE and stained for 45 min with colloidal Coomassie Brilliant Blue. The stacking gel bands were predigested and digested as previously described and 1 μl of a mixture of heavy-labelled peptides was added to each sample before LC-SRM analyses.

All separations were carried out on an Agilent 1100 Series HPLC system (Agilent Technologies). For each analysis, the sample was loaded into a trapping column ZORBAX 300SB-C18 MicroBore Guard 5 μm, 1.0 × 17 mm^2^ (Agilent Technologies) at 50 μl min^−1^ with aqueous solution containing 0.1% (v/v) formic acid and 2% acetonitrile. After 3 min trapping, the column was put on-line with a ZORBAX 300SB-C18 3.5 μm, 0.3 × 150 mm^2^ column (Agilent Technologies). Peptide elution was performed at 5 μl min^−1^ by applying a linear gradient of solvent A (water with 2% acetonitrile and 0.1% (v/v) formic acid) and B (acetonitrile with 0.1% (v/v) formic acid), from 8 to 42% solvent B over 30 min followed by a washing step (2 min at 90% solvent B) and an equilibration step (13 min at 8% solvent B). The isolation width for both Q1 and Q3 was set to 0.7 *m*/*z* unit. The collision energy was experimentally optimized by testing nine values centred on the calculated value from the one given by the supplier. Time-scheduled SRM method targeted the pairs of isotopically labelled peptides/endogenous peptides in ±5 min retention time windows within a cycle time of 3 s. Mass data collected during LC-SRM were processed with the Skyline open-source software package 3.1.1 (ref. 55). Area intensity ratios of the heavy and the light forms of each peptide were manually checked. The endogenous peptide amount calculation was performed by using the most suitable calibration curve. The mass spectrometry and LC-SRM data were deposited to the ProteomeXchange^56^ Consortium via PRIDE^57^ partner repository, and the peptide atlas SRM experiment library (PASSEL), respectively.

### Production of recombinant glutathione S-transferase (GST)-*Tg*HP12 and specific antibodies

The DNA corresponding to C-terminal sequence of 254 amino acids long from 1,051 to 1,812 nucleic acid was amplified by PCR using the following primers: sense 5′-CCGGGGATCCGTAGAAAAGCCTACAACGGTGGGG-3′, and antisense, 5′-CCGGGCGGCCGCTCACAATCTGTCAAGTCTTCCTCCAGTC-3′. The amplified DNA was purified and cloned in frame into pGEX6P3. After verification by sequencing, the plasmid was used to transform *E. coli* BL21 for recombinant protein expression. Protein was purified by GST column and 100 μg of protein was used to immunize one Wistar (RjHan:WI) rat (Janvier Labs, France) using complete Freund adjuvant. The rat was challenged three times with 50 μg of protein and incomplete Freund adjuvant before bleeding 10 days after the last boost and the serum was purified.

### GST-pull down experiments

Recombinant Rab5B, Rab7 and Rab11B proteins were fused to GST using pGEX6P3. After transformation of BL21 *E. coli*, lysates were incubated to Glutathione-beads and washed four times with buffer A: 50 mM Tris-HCl pH 7.5, 0.5M NaCl, 270 mM sucrose 1 mM EGTA, 1 mM EDTA, 1% Triton X-100 and 0.5 mM PMSF) and washed six time with buffer A without Triton X-100. Total *Tg*Vps35-HA and *Tg*Vps26-HA knock-in parasite extracts were prepared from 10^9^ tachyzoites that were lysed with buffer B: 10 mM HEPES pH 7.9 1.5 mM MgCl_2_, 10 mM KCl, 0.5 mM DTT, 0.1 mM EDTA, 0.65% NP40 and 0.5 mM PMSF. The parasite lysate (equivalent to 2.0 × 10^8^ tachyzoites) were added in pull down buffer containing 50 mM Tris-HCl pH 7.5, 150 mM NaCl and 0.5 mM PMSF and incubated with beads containing 2 μg GST-Rab5, GST-Rab7, GST-Rab11B or GST alone in the presence of 1 mM GTP or GDP overnight at 4 °C. Precipitants were washed three time with the pull down buffer containing 0.1% Triton X-100 and eluted by Laemmli buffer and analysed by western blots, which were probed with anti-HA antibodies and anti-GST.

### Statistical analysis

All data were analysed with Graph Pad Prism software. A Student's *t*-test was used for statistical analysis. The Mann–Whitney test was also used for analysis of mice survival curves.

## Additional information

**Accession codes**: The mass spectrometry proteomics data collected during nanoLC-MS/MS analyses have been deposited to the ProteomeXchange Consortium via PRIDE partner repository with the data set identifier PXD003603. The LC-SRM data have been deposited in the Peptide Atlas SRM Experiment Library (PASSEL) with the data set identifier PASS00824.

**How to cite this article:** Sangaré, L. O. *et al*. Unconventional endosome-like compartment and retromer complex in *Toxoplasma gondii* govern parasite integrity and host infection. *Nat. Commun.* 7:11191 doi: 10.1038/ncomms11191 (2016).

## Supplementary Material

Supplementary Information Supplementary Figures 1-4, Supplementary Tables 1-5 and Supplementary References

Supplementary Data 1 List of relevant proteins identified by proteomic analysis using nanoLC-MS/MS.

Supplementary Data 2 Absolute quantitation of core retromer components by LC-SRM.

Supplementary Data 3 Complete list of proteins and the peptides identified by mass spectrometry using Obitrap Mass spectrometer.
