# Supplementary material for: Unconventional endosome-like compartment and retromer complex in Toxoplasma gondii govern parasite integrity and host infection
Source: Nat Commun. 2016 Apr 11;7:11191. doi: 10.1038/ncomms11191 (PMC4831018; doi:10.1038/ncomms11191)
Supplement: Supplementary Information — Supplementary Figures 1-4, Supplementary Tables 1-5 and Supplementary References [file ncomms11191-s1.pdf]

SUPPLEMENTARY INFORMATION

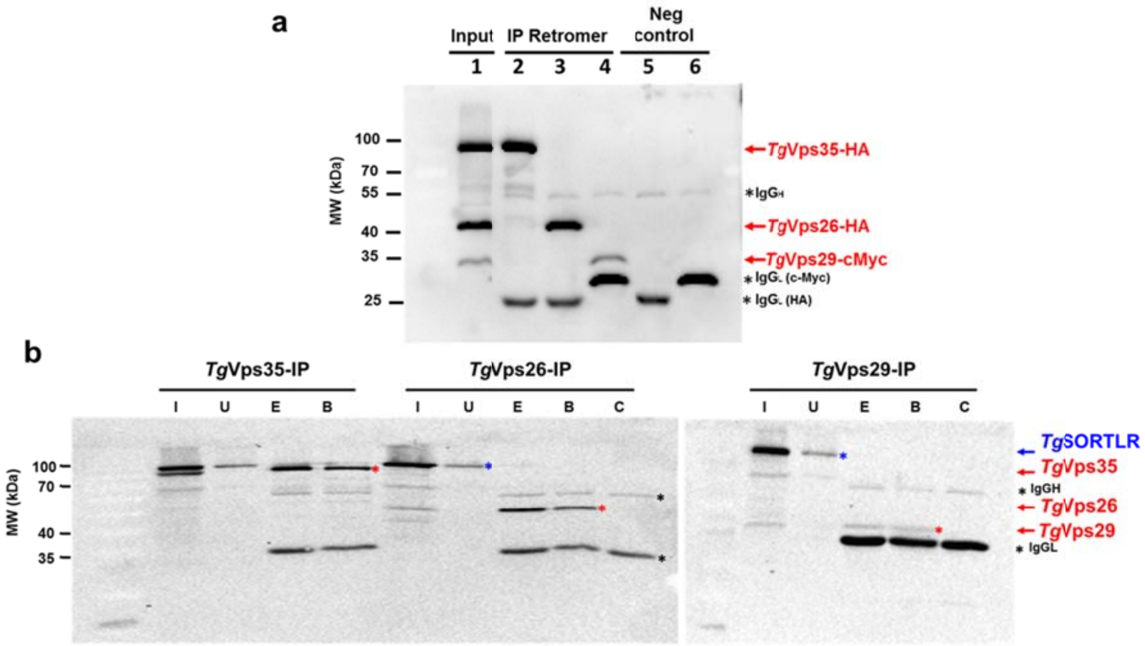

Supplementary Figure 1: Immunoblots of co-immunoprecipitates of *Tg*Vps35-HA, *Tg*Vps26-HA and *Tg*Vps29-cMyc

These full blots corresponding to Figure 1a and 1b were probed with anti-HA, anti-cMyc and anti-TgSORTLR antibodies (see list of antibodies in Supplementary Table 5). Protein markers (kDa) were also shown on left. IgG_h means heavy chain of IgG, IgG_L means light chain of IgG.

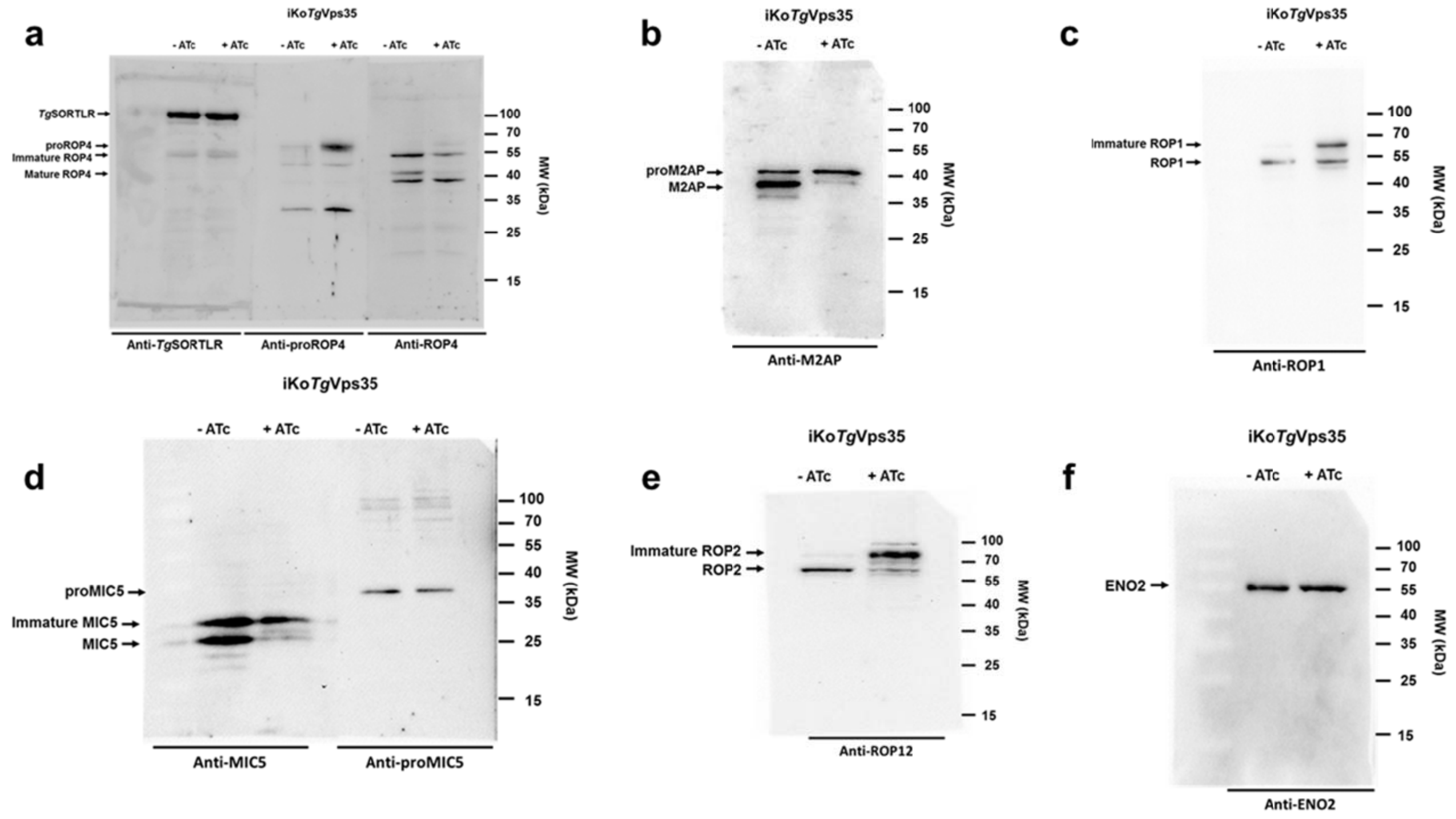

Supplementary Figure 2: Immunoblots of iKo*Tg*Vps35 mutant

These full blots corresponding to Figure 6 were probed with specific anti-ROP1, ROP2, proROP4, ROP4, M2AP, proMIC5 and MIC5 antibodies (see list of antibodies in Supplementary Table 5) after 48h post-infection in the presence or absence of ATc. *Tg*SORTLR and ENO2 protein were used as loading protein control. Protein markers (kDa) were also shown on right.

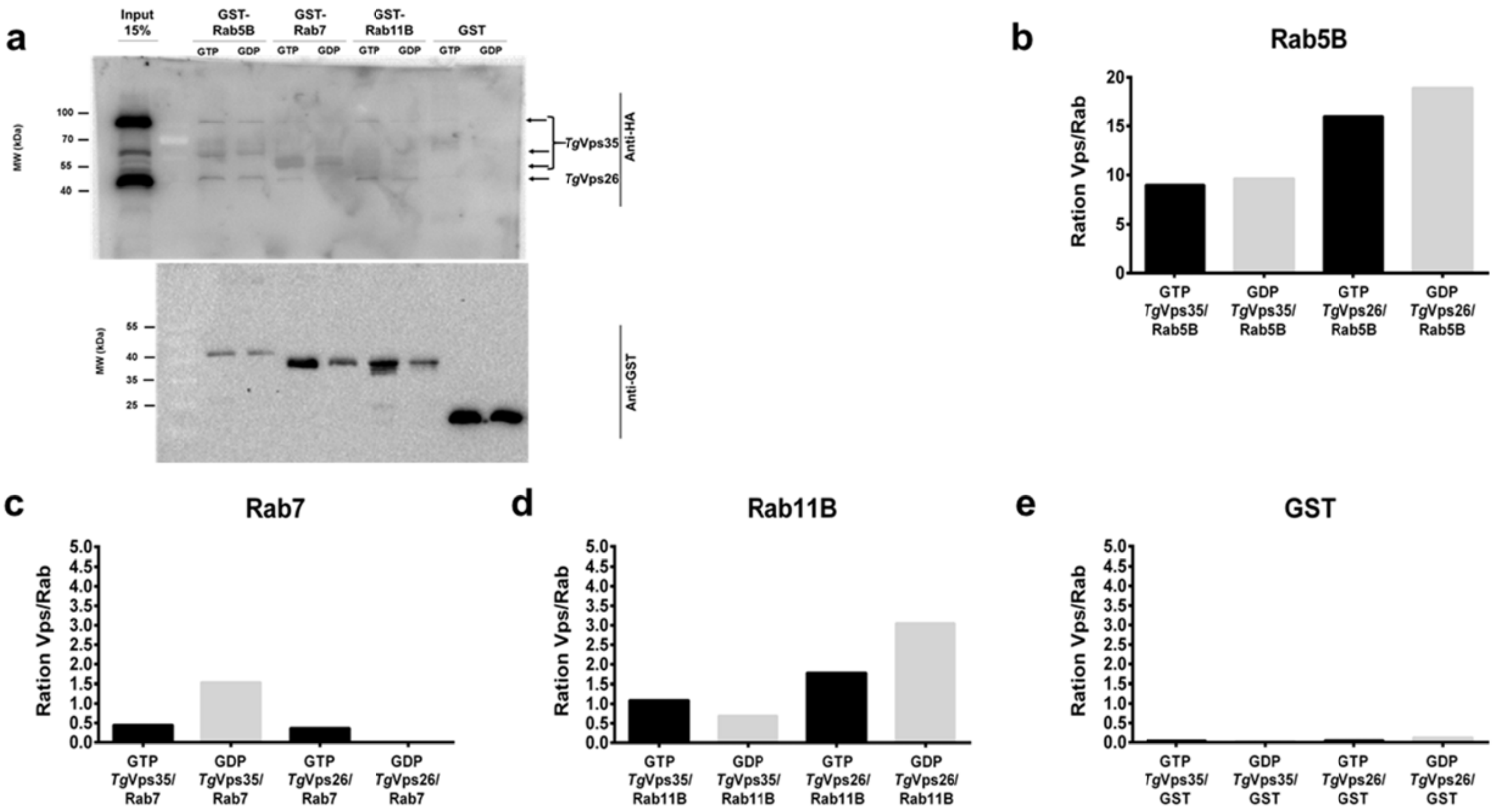

Supplementary Figure 3: Rab7 specifically interacts with the core retromer component TgVps26

(**a**) Immunoblots of Rab-GST pull-downs reveal that only Rab7 interacts with *Tg*Vsp26 protein in a GTP-dependent manner, while Rab5B and Rab11B bind non-specifically to *Tg*Vsp35 and *Tg*Vps26 in the presence of both GTP and GDP (upper panel). The lower panel corresponds to immunoblots demonstrating that equal amount of Rab proteins have been used for the GST-pull down

experiments using anti-GST antibodies. Input corresponds to the total detergent protein extract containing *Tg*Vps35-HA and *Tg*Vps26-HA stained with anti-HA antibodies. Molecular weights (kDa) are shown on left.

(**b-d**) Quantification showing the ratio of the intensity of *Tg*Vsp35 and *Tg*Vsp26 detected in the upper panel of (**a**) versus the signal of the corresponding Rab5B, Rab7, and Rab11B proteins used for the GST pull-down. (**e**) GST alone was used as a negative control for the GST pull down experiments described above.

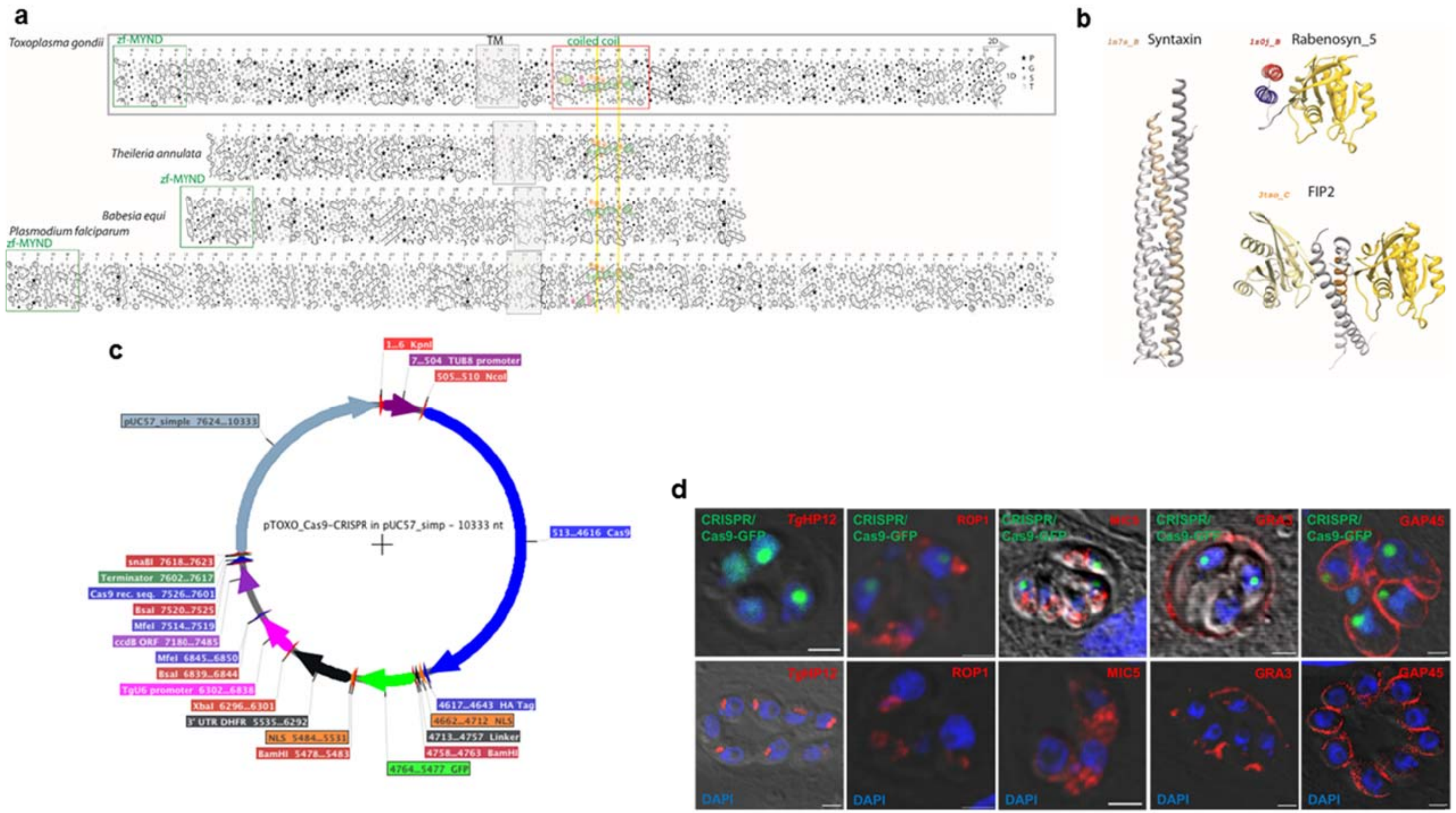

Supplementary Figure 4: Bioinformatics and genetic analyses of *Tg*HP12

(**a**) Hydrophobic cluster analysis[1] was used to identify *Tg*HP12 homologs. The sequences are shown on a duplicated helical net, which displays the contours of the hydrophobic amino acids (V I L M F Y W) that form clusters corresponding to the internal faces of the regular secondary structures. A conserved helical region (boxed) is highlighted on the entire *Tg*HP12 sequences following a

putative transmembrane (TM) segment. Cluster similarities are indicated in green, whereas sequence identities and similarities are shown in orange and pink, respectively.

(**b**) Experimental 3D structures of proteins are shown to include segments (colored), which share some sequence similarities with the conserved helical region of *Tg*HP12. Using the profile-profile comparison tool HH-PRED[2], potential relationships of the *Tg*HP12 helical region were highlighted with two helical structures present in rabenosyn-5 and FIP2, which are known to be involved in the interaction with the Rab switch regions[3,4]. The colored segments correspond to those highlighted on the sequence alignment shown in Figure 8a. Rab proteins are shown in yellow (at right). Another putative similarity was also established with the coiled-coil region of syntaxin, which shares typical heptad repeats with *Tg*HP12[5].

(**c**) Map of plasmid used for *Tg*HP12 disruption by CRISPR/Cas9 system.

(**d**) Confocal images of intracellular tachyzoites in which the *Tg*HP12 gene was disrupted by transient transfection of CRISPR/Cas9. These parasites were directly observed for GFP expression or stained with anti-ROP1, anti-MIC5, anti-GRA3, or anti-GAP45 antibodies (upper panels). As negative controls (lower panels), parental tachyzoites were not transfected and observed for GFP or stained with the same antibodies. Note that there is no phenotypic difference between Cas9-mediated disruption of *Tg*HP12 and the parental parasites for biogenesis of rhoptries, micronemes, and dense granules. Bars=2 µm.

	IP *Tg*Vps26		IP *Tg*Vps29		IP *Tg*Vps35	
**Protein Name**	**Quantity (fmol)**	**Stoichiometry**	**Quantity (fmol)**	**Stoichiometry**	**Quantity (fmol)**	**Stoichiometry**
*Tg*Vps26	25 ± 2	1.0	39 ± 12	0.3	21.4 ± 0.9	0.7
*Tg*Vps29	8 ± 1	0.3	138 ± 34	1.0	12 ± 2	0.4
*Tg*Vps35	31.4 ± 0.4	1.3	54 ± 2	0.4	29.6 ± 0.4	1.0

Supplementary Table 1: Absolute quantification of each retromer component.

Quantification was performed using LC-SRM and isotope-labeled reference peptides. Protein ratios from duplicate experiments were averaged, and protein stoichiometry was shown. Also see more detail in Supplementary Data 2.

Accession	Name	MW [kDa]	∑coverage (%)	∑Unique Peptides	∑Peptides
TGGT1_294220	*Tg*HP12	62.2	78	53	53
TGGT1_290160	*Tg*SORTLR	113.4	35	32	32
TGGT1_263500	*Tg*Vps26	42.2	8	2	2
TGGT1_242660	*Tg*Vps35	95.5	7	5	5
TGGT1_252490	*Tg*Vps29	22.3	6	1	1

Supplementary Table 2: **Identification of *Tg*HP12 by mass spectrometry**

Co-immunoprecipitates of *Tg*HP12 using anti-HA beads and total detergent protein extract from the knock-in *Tg*HP12-HA parasites were analyzed by mass spectrometry to reveal the presence of *Tg*Vps35, *Tg*Vps29, *Tg*Vps26, and *Tg*SORTLR that were specifically pulled down as compared to the naïve sera. Also see more detail in Supplementary Data 3.

Name	5’ Sequence 3’	Restriction sites
iKO_*Tg*Vps35_3’_F	CCGGGGATCCATGtacccatacgatgttccagattacgctATTATGGAACACGATCAAGAAAAACTGCTGGA	BamHI
iKO_*Tg*Vps35_3’_R	CCGGCCTAGGAATTCAAAGAAGTGACTGCAAAGAGAGATCCA	AvrII
iKO_*Tg*Vps35_5’_F	CCGGCATATGTGGTGTGGCTCGTTGAAATTCTCC	NdeI
iKO_*Tg*Vps35_5’_R	CCGGCATATGTCTCGAGCACTTTGGGAGACTCCAA	NdeI
iKO_test1_*Tg*Vps35_F	GGCATCTGCGAGACCTGCACCCAG	none
DHFR-int_R	GGCGTTGAATCTCTTGCCGACTGTGGAGAGGGAAGTCC	none
compl*Tg*Vps35prom_F	ccggGGCGCGCCGATTGGTGTGGCTCGTTGAAATTCTCCTC	AscI
compl*Tg*Vps35prom_R	ccggGGCGCGCCTCTCGACAACTTGGGAGACTCCAACAG	AscI
complFL*Tg*Vps35_F	ccggGGCGCGCCgagcagaagctgatctcagaggaggacctgATTATGGAACACGATCAAGAAAAACTGCTGG	AscI
complFL*Tg*Vps35_R	ccggCCCGGGCTAAGATGTTGAAACACTGATTTCATTCAGTCCAGT	XmaI
KI_*Tg*Vps35HA_F	TACTTCCAATCCAATTTAATGCCAAGAGTGGGTGTCACAGTTACCTGCC	None
KI_*Tg*Vps35HA_R	TCCTCCACTTCCAATTTTAGCAGATGTTGAAACACTGATTTCATTCAGTCCAGTGAGTCC	None
KI_*Tg*Vps26HA_F	TACTTCCAATCCAATTTAATGCCGCGGCTGTCTCTGCATAGGTG	None
KI_*Tg*Vps26HA_R	TCCTCCACTTCCAATTTTAGCCCCGATCTTCTTCCTCCACATTGTGAT	None
KI_*Tg*Vps29myc_F	TACTTCCAATCCAATTTAATGCCCCGATGCGGCCGAGCGGTCAAAAA	None
KI_*Tg*Vps29myc_R	TCCTCCACTTCCAATTTTAGCTTTCTCGGCGGAGCTGGCAGCGTC	None
KI_HP12myc_F	TACTTCCAATCCAATTTAATGCATGGCAACGATGGTCACCTGCCAG	None
KI_HP12myc_R	TCCTCCACTTCCAATTTTAGCCAATCTGTCAAGTCTTCCTCCAGTCA	None
KI_HP03myc_F	TACTTCCAATCCAATTTAATGCGCTGGCTGGCGCACGAAACCTCCGA	none
KI_HP03myc_R	TCCTCCACTTCCAATTTTAGCAGCGGAGTCTTGCGGTGGCGTCACC	none
Recomb_HP12_F	CCGGGGATCCGTAGAAAAGCCTACAACGGTGGGG	BamHI
Recomb_HP12_R	CCGGGCGGCCGCTCACAATCTGTCAAGTCTTCCTCCAGTC	NotI

Supplementary Table 3: Complete list of primers used in this study

iKo, inducible conditional knock-out; KI, knock-in; Comp-, complementation; F, forward primer; R, reverse primer; Recomb, primer for recombinant protein.

Name	Linearisation site	Purpose
pDTS4 iKO*Tg*Vps35	ApaI	Knock Out
pUPRT FL*Tg*Vps35	None	Knock Out
pLIC-HA-DHFR *Tg*Vps35	EcoRV	Knock In
pLIC-HA-DHFR *Tg*Vps26	EcoRV	Knock In
pLIC-Cmyc-DHFR *Tg*Vps29	MfeI	Knock In
pLIC-Cmyc-TUB5CAT *Tg*HP03	BstBI	Knock In
pLIC-Cmyc-TUB5CAT *Tg*HP12	EcoRV	Knock In
pGEX-*Tg*HP12-Cter	None	Recombinant protein

Supplementary Table 4: Complete list of plasmids used in this study

These plasmids were used for parasite knock out, knock in or recombinant protein expression. The restriction enzymes used to linearize these plasmids are indicated otherwise stated.

Name	Species	Dilution (Immunoflorescence or IFA)	Dilution (Western Blot)	Origin
Anti-HA	Rabbit	1/500	1/500	Cell Signaling
Anti-HA	Rat	1/200	1/500	Roche
Anti-cMyc	Mouse	1/500	1/500	Pierce (Thermo)
Anti-*Tg*SORTLR	Rat	1/700	1/700	Tomavo Lab
Anti-MIC2	Mouse	1/500		
Anti-proM2AP	Rabbit	1/500	1/500	Carruthers Lab
Anti-M2AP	Rabbit	1/500	1/500	Carruthers Lab
Anti-proMIC5	Rabbit	1/200	1/500	Carruthers Lab
Anti-MIC5	Rat	1/500	1/500	Carruthers Lab
Anti-ROP1	Mouse	1/500	1/500	Dubremetz Lab
Anti-ROP2-3	Mouse	1/500		Dubremetz Lab
Anti-proROP4	Rabbit	1/500	1/500	Gary Ward Lab
Anti-ROP4	Rabbit	1/500	1/500	Dubremetz Lab
Anti-GRA1	Mouse	1/500	1/500	Dubremetz Lab
Anti-GRA3	Mouse	1/500	1/1000	Dubremetz Lab
SAG1	Mouse	1/200		Tomavo Lab
Anti-CPL	Rabbit	1/200		Carruthers Lab
Anti-VP1	Rabbit	1/400		Carruthers Lab
Anti-HP12	Rat	1/500	1/500	Tomavo Lab

Supplementary Table 5: Complete list of antibodies used in this study

The source, origin and dilution of each antibody used for western blots or immunofluorescence assays are indicated.

SUPPLEMENTARY REFERENCES

1. Callebaut, I., *et al*. Deciphering protein sequence information through hydrophobic cluster analysis (HCA): current status and perspectives. *Cell Mol Life Sci.* **53,** 621-645 (1997).

2. Söding, J., Biegert, A. & Lupas, A. N. The HHpred interactive server for protein homology detection and structure prediction. *Nucleic Acids Res*. **33,** W244-248 (2005).

3. Eathiraj, S., Pan, X., Ritacco, C. & Lambright, D. G. Structural basis of family-wide Rab GTPase recognition by rabenosyn-5. *Nature* **436,** 415-419 (2005).

4. Lall, P., *et al*. Structural and functional analysis of FIP2 binding to the endosome-localised Rab25 GTPase. *Biochim. Biophys. Acta*. **1834,** 2679-2690 (2013).

5. Ernst, J. A. & Brunger, A. T. High resolution structure, stability, and synaptotagmin binding of a truncated neuronal SNARE complex. *J. Biol. Chem.* **278,** 8630-8636 (2003).
